# TWIST1 interacts with β/δ-catenins during neural tube development and regulates fate transition in cranial neural crest cells

**DOI:** 10.1242/dev.200068

**Published:** 2022-08-08

**Authors:** Jessica W. Bertol, Shelby Johnston, Rabia Ahmed, Victoria K. Xie, Kelsea M. Hubka, Lissette Cruz, Larissa Nitschke, Marta Stetsiv, Jeremy P. Goering, Paul Nistor, Sally Lowell, Hanne Hoskens, Peter Claes, Seth M. Weinberg, Irfan Saadi, Mary C. Farach-Carson, Walid D. Fakhouri

**Affiliations:** 1 Center for Craniofacial Research, Department of Diagnostic and Biomedical Sciences, School of Dentistry, University of Texas Health Science Center at Houston, Houston, TX 77054, USA; 2 Department of Bioengineering, Rice University, Houston, TX 77005, USA; 3 Department of Pathology and Immunology, Baylor College of Medicine, Houston, TX 77030, USA; 4 Department of Anatomy and Cell Biology, The University of Kansas Medical Center, Kansas City, KS 66160, USA; 5 Centre for Regenerative Medicine, Institute for Stem Cell Research, School of Biological Sciences, University of Edinburgh, Little France Drive, Edinburgh EH16 4UU, UK; 6 Department of Electrical Engineering, ESAT/PSI, KU Leuven, Leuven 3001, Belgium; 7 Medical Imaging Research Center, UZ Leuven, Leuven 3000, Belgium; 8 Department of Human Genetics, KU Leuven, Leuven 3000, Belgium; 9 Center for Craniofacial and Dental Genetics, Department of Oral and Craniofacial Sciences, University of Pittsburgh, Pittsburgh, PA 15219; 10 Department of Human Genetics, University of Pittsburgh, Pittsburgh, PA 15260, USA; 11 Department of Pediatrics, McGovern Medical School, University of Texas Health Science Center at Houston, Houston, TX 77030, USA

**Keywords:** Mouse genetics, Neural tube closure, Epithelial-to-mesenchymal transition, Neural tube explants, Cell delamination, Cell migration

## Abstract

Cell fate determination is a necessary and tightly regulated process for producing different cell types and structures during development. Cranial neural crest cells (CNCCs) are unique to vertebrate embryos and emerge from the neural plate borders into multiple cell lineages that differentiate into bone, cartilage, neurons and glial cells. We have previously reported that *Irf6* genetically interacts with *Twist1* during CNCC-derived tissue formation. Here, we have investigated the mechanistic role of *Twist1* and *Irf6* at early stages of craniofacial development. Our data indicate that TWIST1 is expressed in endocytic vesicles at the apical surface and interacts with β/δ-catenins during neural tube closure, and *Irf6* is involved in defining neural fold borders by restricting AP2α expression. *Twist1* suppresses *Irf6* and other epithelial genes in CNCCs during the epithelial-to-mesenchymal transition (EMT) process and cell migration. Conversely, a loss of *Twist1* leads to a sustained expression of epithelial and cell adhesion markers in migratory CNCCs. Disruption of TWIST1 phosphorylation *in vivo* leads to epidermal blebbing, edema, neural tube defects and CNCC-derived structural abnormalities. Altogether, this study describes a previously uncharacterized function of mammalian *Twist1* and *Irf6* in the neural tube and CNCCs, and provides new target genes for *Twist1* that are involved in cytoskeletal remodeling.

## Introduction

Craniofacial morphogenesis requires precise interaction between multiple genes and gene environments that are essential for cell fate induction, specification and differentiation (Chai and Maxson, 2006; Nomura and Li, 1998; Padmanabhan and Ahmed, 1997). Disruption of the precisely organized morphogenesis leads to craniofacial disorders, which are the second most common congenital birth defects in humans (Hoyert et al., 2006; Joshi et al., 2014; Murray et al., 2007; Parker et al., 2010). During embryogenesis, signaling molecules from the notochord and mesoderm induce the formation of neuroectoderm. The junctions between non-neural and neural ectoderm are called the neural plate borders, which form distinct domains through inductive interactions with the adjacent cells and the underlying mesoderm (Groves and LaBonne, 2014; Litingtung and Chiang, 2000; Millonig et al., 2000). The CNCCs emerge from the neural fold borders of the midbrain and hindbrain regions, and migrate as unipotent and multipotent mesenchymal cells towards the frontonasal processes (Achilleos and Trainor, 2012; Zhang et al., 2021). The CNCCs give rise to the bone, cartilage, neuron and glial cells of the orofacial skeletal and peripheral nervous systems (Groves and LaBonne, 2014; He et al., 2014; Le Douarin et al., 2004; Noden and Trainor, 2005).

*Twist1* encodes a transcription factor that belongs to the basic helix-loop-helix (bHLH) B-family. It was first identified in *Drosophila melanogaster* where its deletion resulted in disruption of mesodermal specification and ventral furrow formation (Lu et al., 2011; Nüsslein-Volhard et al., 1984; Simpson, 1983). In vertebrates, *Twist1* is expressed first at the gastrulation stage in the primitive streak and then in the adjacent mesodermal cells (Füchtbauer, 1995; Stoetzel et al., 1995). At the neurulation stage, mammalian *Twist1* is detected in the paraxial and lateral mesoderm, and the migratory CNCCs (Bildsoe et al., 2016; Füchtbauer, 1995; Ota et al., 2004; Soo et al., 2002). *Twist1* expression is maintained in the CNCC-derived mesenchyme of the frontonasal and pharyngeal processes (Bildsoe et al., 2009; Füchtbauer, 1995). In humans, mutations in *TWIST1* cause Saethre-Chotzen syndrome, which is characterized by craniosynostosis and cleft palate, Sweeney-Cox syndrome and Robinow-Sorauf syndrome (Kunz et al., 1999; Takenouchi et al., 2018; Seto et al., 2007). In mice, *Twist1* is crucial for neural tube closure and for CNCC-derived craniofacial bone and cartilage formation (Chen et al., 2007; Bildsoe et al., 2013). TWIST1 has been shown to promote cell survival and proliferation of migratory CNCCs during craniofacial development (Bildsoe et al., 2013). Although both processes involve gaining potency and motility, the *in vivo* molecular function of TWIST1 as a master regulator of epithelial-to-mesenchymal transition (EMT) in CNCCs was thus far only described in *Xenopus* (Lander et al., 2013).

TWIST1 is a phosphoprotein with multiple residues that are phosphorylated by several kinases according to numerous cancer studies (Lu et al., 2011; Vichalkovski et al., 2010; Xue and Hemmings, 2012). TWIST1 phosphorylation is crucial for regulating its homo- and heterodimerization with other factors to control multiple cellular activities (Firulli and Conway, 2008; Hong et al., 2011; Lu et al., 2011; Vichalkovski et al., 2010). TWIST1 interacts with other proteins, such as TCF3 and chromatin remodeling factors, to guide CNCC migration during craniofacial development (Firulli et al., 2005; Fan et al., 2021). Misregulation of TWIST1 dimerization with HAND2 is caused by mutations of phosphoresidues in the PKA consensus domain or in the bHLH domain, which have been reported to be associated with Saethre-Chotzen syndrome (Firulli et al., 2005; El Ghouzzi et al., 1997). Mutations within the TWIST-box domain are also associated with the isolated form of craniosynostosis (Seto et al., 2007). TWIST1 is also expressed in cardiomyocytes and is repressed in patients with dilated cardiomyopathy. Knockdown of *Twist1* in primary rat cardiomyocytes that are stimulated using phenylephrine/isoprenaline causes significant reduction in cell size (Baumgarten et al., 2013). Overexpression of *Twist1*^*T125D/S127D*^ phosphomimetic alleles (equivalent to T121/S123 in human) in cardiomyocytes resulted in disrupted cell remodeling and heart formation of transient transgenic mouse embryos (Lu et al., 2011).

Our previous work demonstrated that the compound heterozygous mice (*Irf6*^*+/*−^; *Twist1*^*+/*−^) have craniofacial abnormalities, i.e. mandibular agnathia, fused maxilla, cleft palate and holoprosencephaly (Fakhouri et al., 2017). Unlike other IRF family members, interferon regulatory factor 6 (*Irf6*) is a broadly expressed transcription factor in the ectoderm and oral epithelium during embryogenesis. In mice, *Irf6* promotes cell differentiation of proliferative epithelial cells, but its function in the neural tube and neural crest cell development is still unknown (Ingraham et al., 2006; Kousa et al., 2019; Richardson et al., 2006). We have recently shown that overexpression of *Irf6* in ectoderm by K14-enhancer leads to exencephaly and failure of peritoneal skin development. Furthermore, the compound heterozygous mice for *Irf6* and *Tfap2a* do not show neural tube defects compared to single *Tfap2a* heterozygous mice (Kousa et al., 2019).

In the current study, we sought to characterize the spatiotemporal expression of *Twist1* and *Irf6* during neural tube formation and their function in CNCCs. We generated multiple stable *Twist1* phospho-incompetent mouse lines to identify the importance of TWIST1 phosphorylation in regulating neural tube and CNCC-derived craniofacial structures. We investigated the molecular role of *Irf6* in regulating the integrity of the neural tube and its relation to AP2α expression. Our data show that TWIST1 protein and mRNA are detected at the apical side of dorsal neuroectodermal cells within expression locations similar to β-catenin and tight junction proteins. Further investigation suggests that TWIST1 is expressed in endocytic compartments at the apical surface and partially overlaps with endocytic markers LRP2 and RAB11b. Meanwhile, cytosolic TWIST1 interacts with β/δ-catenin during neural tube formation. We show that *Twist1* is crucial to promote cell fate transition of pre-EMT neuroectodermal cells, and loss of *Twist1* in neuroectoderm causes ectopic expression of IRF6 and E-cadherin in delaminated CNCCs. We demonstrate that three highly conserved phospho-residues in TWIST1 are crucial for its *in vivo* function in the neural tube and in CNCC-derived structures. Finally, our data shows that TWIST1 regulates *Specc1l* expression during CNCC development to possibly control cytoskeletal remodeling in CNCCs.

## Results

### Spatiotemporal expression of TWIST1 and IRF6 during neural tube development

**Fig. 1:**
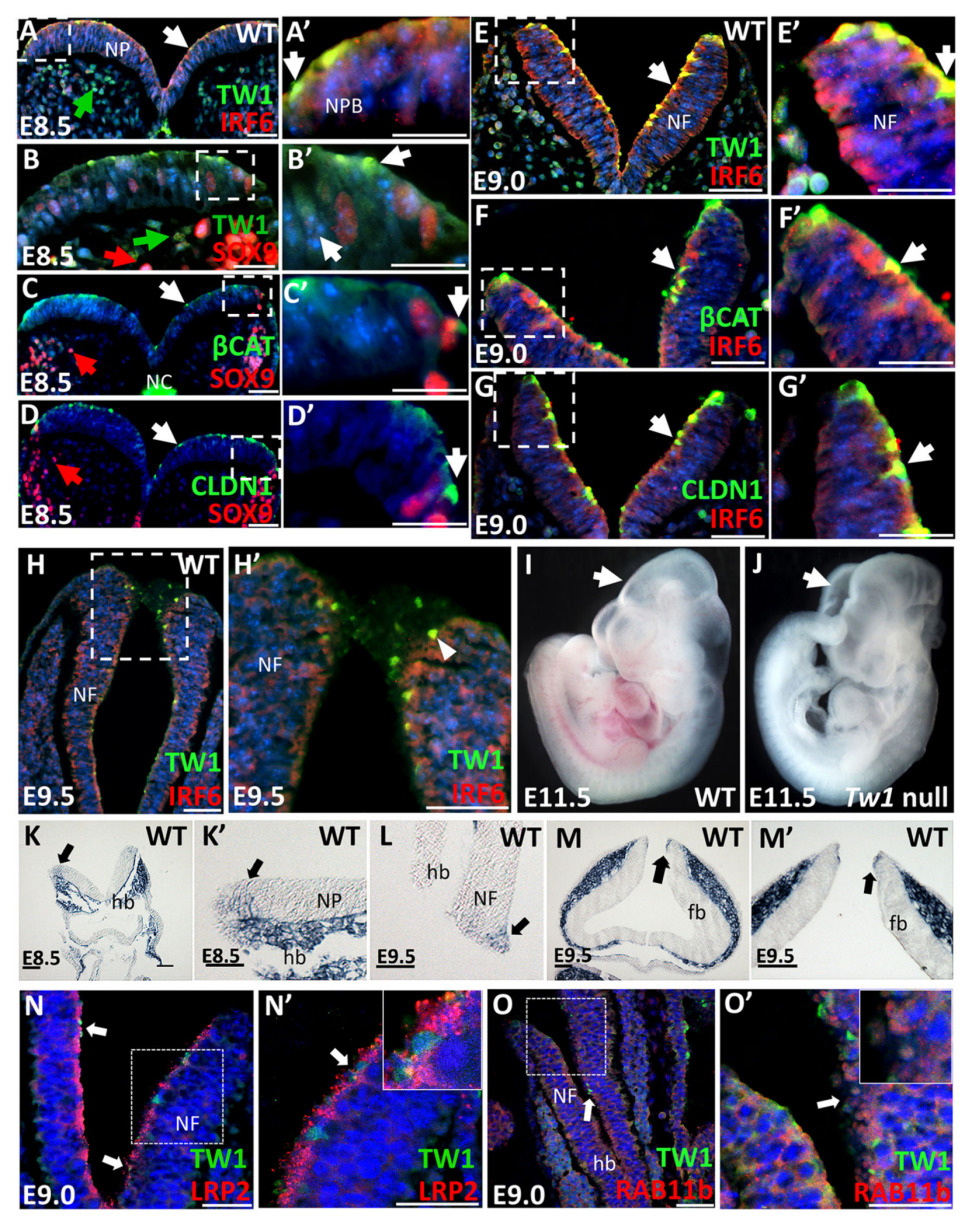
Spatiotemporal expression of TWIST1 and IRF6 in neural folds. (A,A′) IRF6 is present mostly on the apical side of the neural plate, with little expression seen in the middle; TWIST1 is expressed in specific locations at the apical side of neural plate (NP) and in the adjacent mesenchymal cells at E8.5. (B,B′) TWIST1 is also expressed in a few cells residing at the neural plate borders and overlaps with SOX9 expression in nucleus of a few cells at E8.5. SOX9 expression indicates CNCCs, and is seen in migrating cells and in the neural plate borders. (C,C′) β-catenin and (D,D′) claudin 1 are strongly expressed in specific locations at the apical side of neural plate. (E-G′) At E9.0, TWIST1 and IRF6 (E,E′), β-catenin (F,F′), and claudin 1 (G,G′) are expressed in similar locations in the neural folds (NFs) as they are at E8.5. IRF6 is mostly expressed in the apical and basal side of the neural plate with little expression seen in the middle. A-G are consecutive sections of a wild-type embryo at each embryonic time point. (A′-G′) Regions outlined in A-G shown at higher magnification. (H,H′) TWIST1 and IRF6 are detected during neural tube closure, and TWIST1 is expressed at the dorsal edges of neural folds (area outlined in H is shown in H′). (I,J) Wild-type embryos have closed neural tubes at E11.5 (I), yet *Twist1*-null neural tube is completely open (J). (K-M′) *Twist1* mRNA is expressed in the dorsal neural folds of hindbrain at E8.5 and E9.5 by *in situ* hybridization. Three biological replicates were used for each embryonic time point in the *in situ* hybridization. (N,N′) Dual immunostaining of TWIST1 in green and the endocytic receptor LRP2 in red show colocalization at the apical surface of neural folds and partial overlap in vesicle structures. (O,O′) Dual immunostaining of TWIST1 in green and the endocytic marker RAB11b in red shows partially overlap in compartment vesicles at the apical side of neural folds. (N′,O′) Regions outlined in N,O are shown at higher magnification. Insets in N′,O′ indicate overlapping regions. For all immunofluorescent staining, four biological replicates were used for each genotype and for each embryonic time point tested. Arrows indicate areas shown at higher magnification in the insets. Scale bars: 50 μm. NP, neural plate; NF, neural fold; hb, hindbrain; fb, forebrain.

To determine the role of *Twist1* and *Irf6* in neural tube formation, we investigated their spatiotemporal expression in wild-type embryos. We performed immunofluorescent staining and *in situ* hybridization in embryos from E8.5 to E9.5. At E8.5, IRF6 was highly expressed in the basal and apical sides of neural plate, with low expression seen in the middle and weak IRF6 expression detected in some cells beneath the neural plate (Fig. 1A,A′). TWIST1 was unexpectedly detected in vesicle-like structures at the apical surface of neural plate (Fig. 1A,A′, white arrow; Fig. S1A-B). Nuclear TWIST1 was weakly expressed in a few cells at the neural plate borders (Fig. 1B,B′, white arrow). Robust nuclear expression of TWIST1 was observed in the mesodermal cells adjacent to the neural plate (Fig. 1A,B, green arrow). At E9.0, most dorsal and ventral cells in neural folds expressed IRF6, while TWIST1 was mostly expressed at the apical side and dorsal edges of neural folds. TWIST1 expression overlapped with IRF6 at the dorsal edges of the neural folds (Fig. 1E,E′, white arrow; Fig. S1C). To understand the role of TWIST1 apical expression, we investigated whether its expression was associated with adherens and tight junction proteins. TWIST1 apical expression in neuroectodermal cells had similar expression patterns to β-catenin (Fig. 1C,C′; Fig. S1C,D) and claudin 1 at E8.5 (Fig. 1D,D′) and E9.0 (Fig. 1F-G′; Fig. S1E). SOX9 was used as a marker of pre-EMT and migratory CNCCs (Fig. 1B-D, red arrow). TWIST1 apical expression was also observed at the dorsal edges of the neural folds immediately before fusion (Fig. 1H-H′, arrowhead). We tested the specificity of the anti-TWIST1 antibodies in *Twist1*-null embryos. Histological staining showed the neural tube closure defects in *Twist1*-null compared with wild-type embryos at E10.5 (Fig. S2A-B′). No signal for TWIST1 protein was detected in the neural tube or mesenchymal cells of a *Twist1*-null embryo compared with a wild type (Fig. S2C-F′). Notably, *Twist1* null embryos had complete neural tube closure defects (Fig. 1J, white arrow) compared with wild-type littermates at E11.5 (Fig. 1I, white arrow). In *Twist1* null embryos, the neural plate invaginated and lateral neural folds elevated towards the dorsal midline but the lateral folds failed to close (Fig. 1J, white arrow). To validate the immunofluorescence data, whole-mount *in situ* hybridization for *Twist1* mRNA showed weak expression at the dorsal edges of the neural plate of E8.5 embryos (Fig. 1K-K′; Figs S3 and S4) and of neural folds of E9.5 embryos (Fig. 1L-M′; Fig. S5). Based on a previous publication reporting the expression of LRP2 and RAB11b, a marker of endocytic cellular membrane vesicles, at the apical surface (Kowalczyk et al., 2021), we investigated whether TWIST1 was expressed in endocytic vesicles of RAB11b compartments. The dual immunofluorescent staining data shows that TWIST1 was expressed in endocytic vesicles at the apical surface of neural folds (Fig. 1N-O′). TWIST1 expression partially overlapped with LRP2 at the apical side of neural tube (Fig. 1N,N′, inlet in N′; Fig. S1A,A′) and with RAB11b in endocytic compartments (Fig. 1O,O′, inlet in N′; Fig. S1B,B′). *Rab11b* mRNA was significantly reduced in hindbrain tissues of *Twist1* conditional knockout (CKO), while no change was detected for *Lrp2* expression (Fig. S6C,D).

**Fig. 2:**
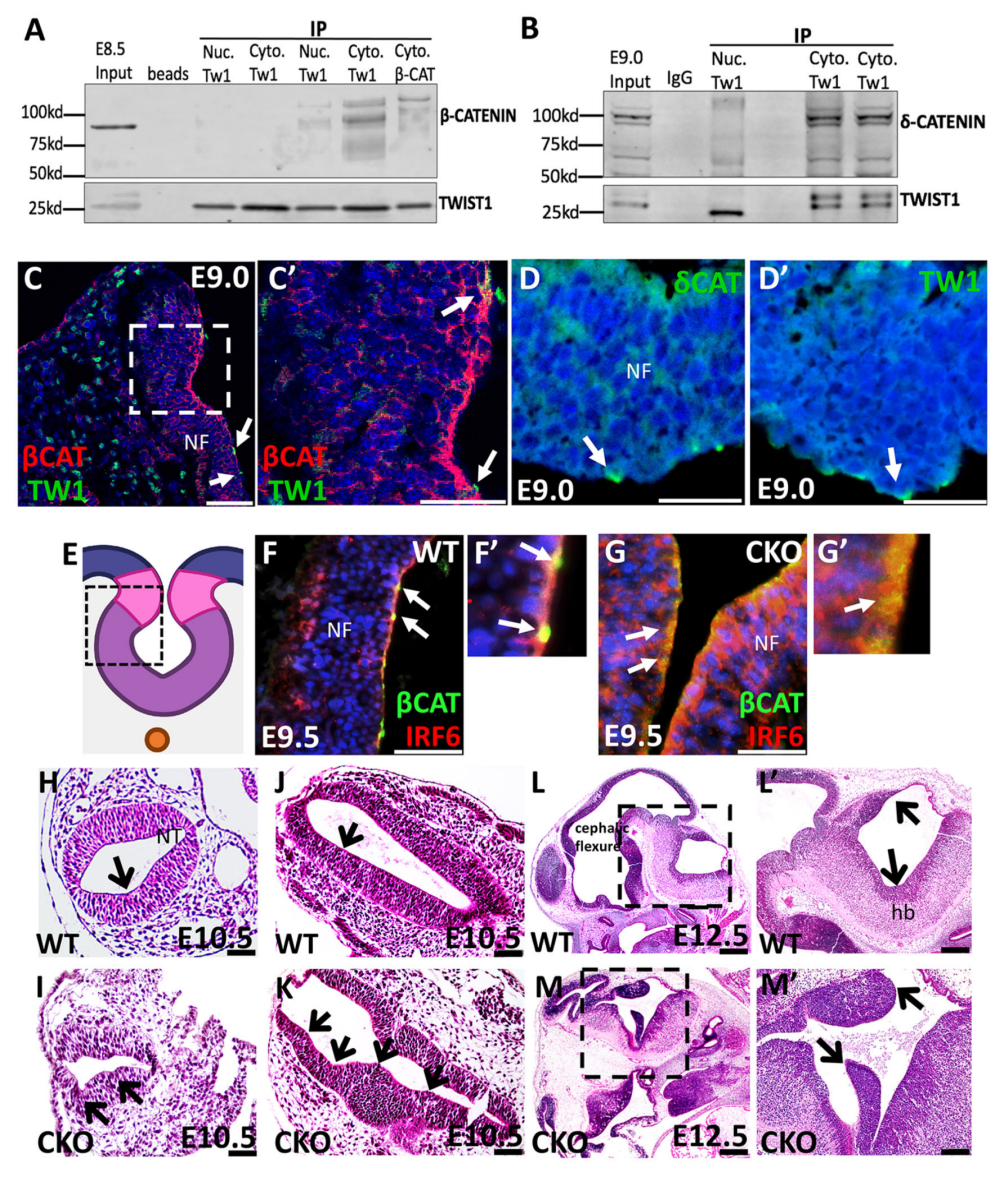
Twist1 interaction with β/δ-catenin proteins. (A,B) The *in vivo* cellular fractionation co-IP assay shows that cytosolic TWIST1 strongly interacts with β-catenin, whereas nuclear TWIST1 weakly interacts with β-catenin at E8.5 (A) and with δ-catenin in the cytosolic fraction (B). TWIST1 protein is detected in the input extract and is enriched in the pulldown extract. Nuclear TWIST1 weakly interacts with β-catenin. (C,C′) The dual immunostaining for TWIST1 and β-catenin shows strong β-catenin expression at the apical and apicolateral side of the dorsal ectodermal cells, while weak TWIST1 expression in vesicle structures overlaps with β-catenin at the apical side of neural folds (NFs) (area outlined in C is shown in C′). Arrows indicate TWIST1 expression overlapping with β-catenin. (D,D′) Arrows indicate δ-catenin and TWIST1 expression in vesicle structures at the apical surface of neural folds. (E) A diagram of the neural folds. The outlined area indicates the regions indicated in Fig. 1F,G. (F-G′) β-Catenin is detected apically in focal points of the dorsal cells of the neural tube (NT) at E9.5 (F,F′) and becomes mostly cytosolic in dorsal cells of *Twist1*^*cko/*−^ (G,G′). Arrows indicate β-catenin expression at the apical surface. (H-K) Hematoxylin and Eosin staining shows a normal neural tube formation is observed in wild type (H, J), whereas *Twist1*^*cko/*−^ embryos have multiple ectopic dorsolateral hinge points and expansion of the neural tube (I,K). Arrows indicate hinge points. (L-M′) Histological staining of an older wild-type embryo shows normal development of the cephalic flexure of the midbrain and hindbrain at E12.5 (L,L′), whereas *Twist1*^*cko/*−^ shows abnormal patterning of the cephalic flexure of midbrain and hindbrain (M,M′, black arrows). (areas outlined in L,M are shown in L′,M′). Scale bars: 50 μm.

To further determine the significance of TWIST1 apical expression, we performed co-immunoprecipitation (co-IP) for TWIST1 to identify the protein interactors at E8.5-E9.0. The *in vivo* cellular fractionation co-IP blots show that the β-catenin and δ-catenin in the cytosolic fraction were pulled down with TWIST1 immunoprecipitation using anti-TWIST1 antibodies (Fig. 2A,B). In contrast, nuclear TWIST1 weakly interacted with β-catenin (Fig. 2A). We also performed dual immunostaining for TWIST1 and β-catenin using rabbit anti-β-catenin antibodies. β-Catenin was detected at the apical-lateral side of all the dorsal neuroectodermal cells (Fig. 2C,C′; Fig. S1F,G). Likewise, TWIST1 and δ-catenin were detected at the apical side of neural folds in a similar pattern of consecutive sections (Fig. 1D,D′; Fig. S1H-I′). Fig. 2E shows neural folds and a dotted-line square marks the areas indicated in Fig. 1F,G. β-Catenin apical expression detected in wild type was diffused in the *Twist1*^*cko/*−^ embryo and became mostly cytosolic when detected using mouse anti-β-catenin antibodies (Fig. 2F-G′). To further determine the impact of *Twist1* loss in neural tube formation, Hematoxylin and Eosin staining was performed in wild-type and *Twist1*^*cko/*−^ embryonic tissues. Normal neural tube formation was observed in wild type (Fig. 2H,J), while *Twist1*^*cko/*−^ embryos had multiple dorsolateral bend points and expansion of the neural tube (Fig. 2I,K). Histological staining of older wild-type embryos showed a normal development of the cephalic flexure of the midbrain and hindbrain at E12.5 (Fig. 2L,L′; Fig. S6F-F″), while *Twist1*^*cko/*−^ showed abnormal patterning of the cephalic flexure of midbrain and hindbrain at E12.5 (Fig. 2M,M′, black arrows; Fig. S6H-H″).

### Tracing CNCC migration in wild type and *Twist1*^*cko/*−^ using *ROSA26*^*Tm1*^; *Wnt1-Cre* and *Wnt1-Cre2* mouse lines

**Fig. 3:**
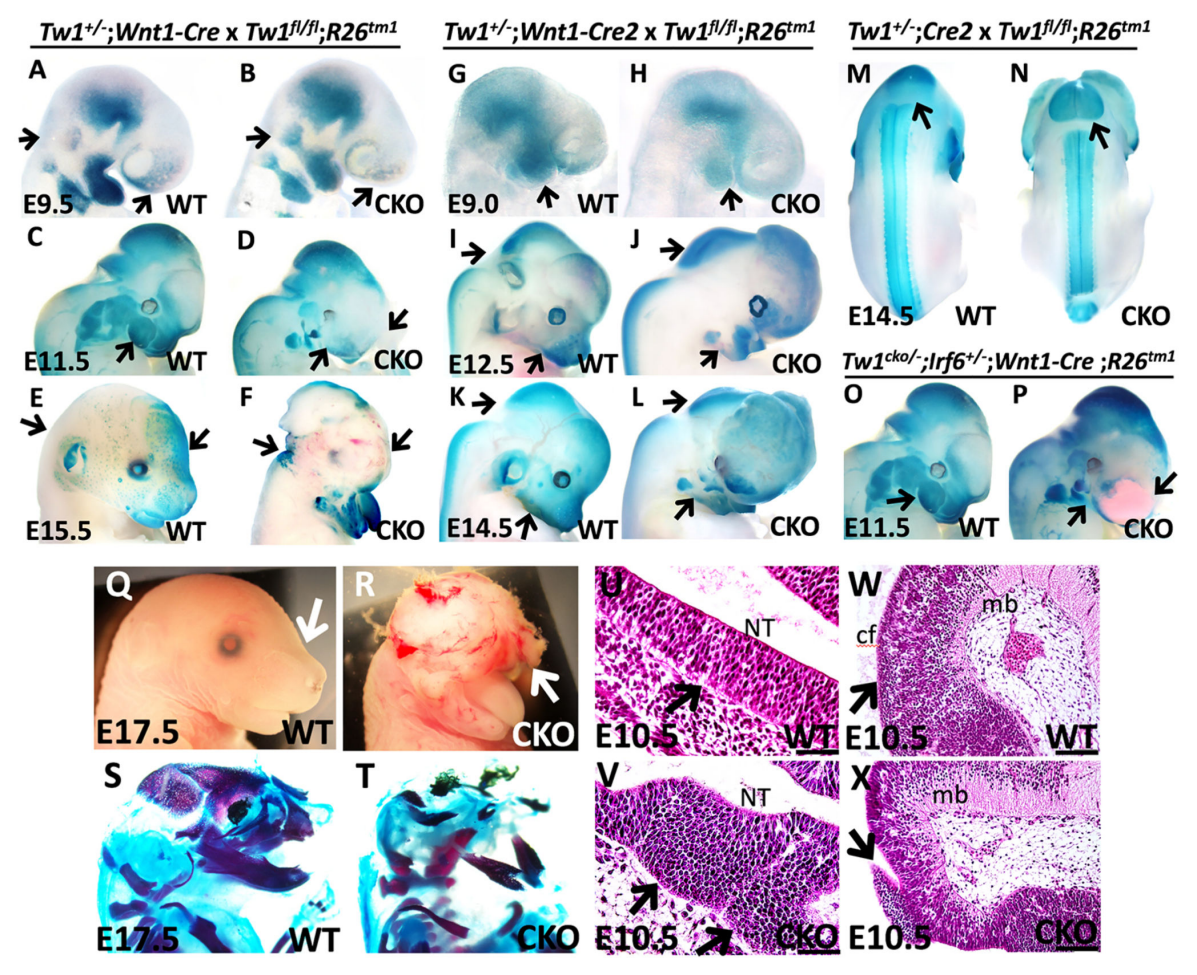
*In vivo* CNCC migration in wild type and *Twist1*^*cko/*−^. (A-P) Alcian Blue and Alizarin Red staining. (A) CNCCs (stained in blue) are detected in the cephalic flexure, frontonasal process and pharyngeal arches of wild type at E9.5. Arrows indicate hindbrain and frontonasal regions. (B) Less staining of migratory CNCCs is observed in frontonasal and pharyngeal arches of *Twist1*^*cko/*−^ embryos, while darker staining is detected in the hindbrain regions. Arrows indicate regions of interest for X-gal staining. (C,D) The mandibles of *Twist1*^*cko/*−^ embryos are smaller than wild type, and the blue staining of CNCCs is weaker in the frontal processes. Arrows indicate the frontonasal and first pharyngeal arch. (E,F) At E15.5, the *Twist1*^*cko/*−^ embryo lacks proper craniofacial development, and displays exencephaly and retained staining at the hindbrain. Arrows indicate hind- and forebrain regions. (G-N) The *R26*^*Tm1*^; *Wnt1-Cre2* cell tracing from E9.5 to E14.5 in wild-type and *Twist1*^cko/−^ embryos show a similar blue staining pattern and phenotype to *Wnt1-Cre*. Arrows indicate regions of interest for X-gal staining. (O,P) *Irf6*^*+/*−^; *Tw1*^*cko/*−^; *Wnt1-cre* embryo shows under-developed mandible, severe forebrain hemorrhage and darker staining at the hindbrain regions compared with wild type. Arrows indicate frontonasal and first pharyngeal arch. (Q,R) Macroscopic images of wild type and *Twist1*^*cko/*−^ at E17.5. Arrows indicate frontonasal regions. (R) *Twist1*^*cko/*−^ embryo shows severe exencephaly and craniofacial abnormalities. (S,T) Skeletal staining of wild type compared with *Twist1*^*cko/*−^ showing loss of most craniofacial bone (red) and nasal cartilage (blue). (U-X) Hematoxylin and Eosin staining. (U) Normal apical and basal layers of neural tube (NT) in wild type. Arrows indicate basal region. (V) *Twist1*^*cko/*−^ neural tube has lateral expansion and partially detached cells at the basal side. Arrows indicate affected basal regions. (W) Wild type shows normal development of cephalic flexure (cf) of midbrain (mb). Arrows indicate brain cortex. (X) In *Twist1*^*cko/*−^, an abnormal midbrain patterning is detected with unorganized protrusions (black arrows). Scale bars: 100 μm.

To determine the role of *Twist1* in CNCCs, we used *Wnt1-Cre* and *Wnt1-Cre2* to conditionally knockout (CKO) *Twist1* in pre-EMT CNCCs. For cell tracing, we used *R26*^*Tm1*^ reporter gene and X-gal whole-mount staining. At E9.5, the blue staining of migratory CNCCs was detected in the cephalic flexure of the midbrain, frontonasal process and pharyngeal arches of wild-type embryos (Fig. 3A), and similar expression pattern was detected in wild type at E11.5 but with stronger staining in the rhombic lips (Fig. 3C). At E15.5, migratory CNCCs were observed in the otic pinna and orofacial regions (Fig. 3E). In mutant *Twist1*^*cko/*−^ embryos, weaker blue staining was observed in the frontonasal and pharyngeal processes, while stronger staining was detected in the mid- and hindbrain regions at E9.5, E11.5 and E15.5, suggesting disruption of migratory CNCCs towards frontal processes (Fig. 3B,D,F). We noticed that the first pharyngeal arch of *Twist1*^*cko/*−^ embryos was smaller at E11.5, and the embryos displayed severe brain abnormalities at E15.5 (Fig. 3D,F). The recently generated *Wnt1-Cre2* mouse line has been used to validate the CNCC-specific deletion by the *Wnt1-Cre* line that has a midbrain developmental abnormality (Lewis et al., 2013). The *Wnt1-Cre2* line showed a similar staining pattern of migratory CNCCs to *Wnt1-Cre* (Fig. 3G, I,K) and the craniofacial phenotype associated with *Twist1*^*cko/*−^ (Fig. 3H,J,L). At E12.5 and E14.5, a dorsal image from the whole-mount staining of wild-type embryos showed blue staining in the hindbrain and in the trunk neural tube (Fig. S6E,E′; Fig. 3M, black arrow). Similarly, blue staining was observed in *Twist1*^*cko/*−^ embryos, showing expanded staining in the midbrain and hindbrain compared with the wild type (Fig. S6G,G′; Fig. 3N, black arrow). The embryos with compound alleles of *Twist1*^*cko/*−^ and *Irf6*^*+/*−^ presented with severe hemorrhaging in the frontonasal process and craniofacial abnormalities compared with wild type (Fig. 3O,P; Fig. S6I-K). Stereomicroscope images of a wild-type embryo showed normal craniofacial development at E17.5 (Fig. 3Q), while a *Twist1*^*cko/*−^ embryo showed severe exencephaly and abnormal frontonasal processes (Fig. 3R). *Twist1*^*cko/*−^ embryos lacked cranial, frontonasal and maxillary bones, and had severe mandibular hypoplasia, as depicted by the skeletal staining (Fig. 3T), compared with wild-type littermate (Fig. 3S). Histological staining of a wild-type embryo showed normal development of the neural tube apical and basal layers of the hindbrain and midbrain at E10.5, respectively (Fig. 3U,W), while *Twist1*^*cko/*−^ showed an expansion of the neural tube and partially detached cells at the basal side, and abnormal patterning of the cephalic flexure of the midbrain (Fig. 3V,X).

### CNCC delamination and migration in WT and *Twist1*^*cko/*−^ neural tube explants

**Fig. 4:**
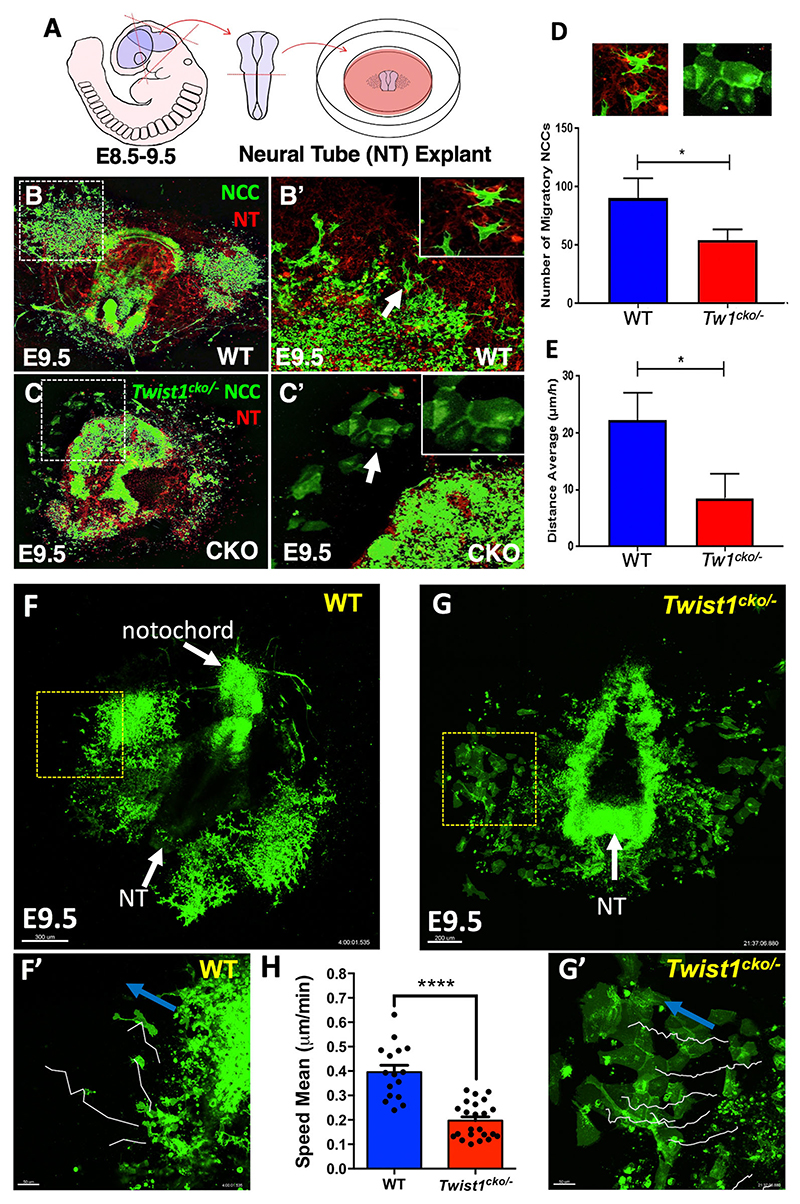
Time-lapse imaging of wild-type and *Twist1*^*cko/*−^ neural tube (NT) explants. (A) Diagram showing the neural tube (NT) explant preparation process. (B-C′) Confocal time-lapse images of NT explants at E9.0. Migratory CNCCs express GFP in wild type (B,B′); *Twist1*^*cko/*−^ neural tube exhibits fewer CNCCs delaminated from the tube and migratory cells maintain their cell-cell adhesion (C,C′). Many pre-EMT CNCCs remained in *Twist1*^*cko/*−^ NT (C,C′). Dashed square and arrow indicate the location of the magnified areas. (D,E) Quantification of total CNCCs and individual cell migration paths (white lines) are shown for wild-type and *Twist1*^*cko/*−^ explants. (F,G) Images show the entire mouse embryo explant for wild type and *Twist1*^*cko*/−^. (F′,G′) The magnified areas indicated by squares in E and F. Blue arrows indicate the direction of cell migration for each explant. (H) Average speed mean migration (µm/min) of wild-type and *Twist1*^*cko/*−^ CNCCs. Neural folds from more than three biological replicates were used for each genotype. *P<0.05, ****P<0.0001. Data are mean±standard error of the sampling distribution. Scale bars: 300 μm for F; 200 μm for G; 50 μm for F′ and G′.

We further investigated why a loss of *Twist1* in pre-EMT neuroectodermal cells led to severely abnormal brain and craniofacial structures, even though a significant number of CNCCs migrated towards the frontonasal and pharyngeal processes. We also asked why the mutant migratory CNCCs did not form craniofacial bone and cartilage (Fig. 3Jj). To answer these questions, we developed a neural tube explant system using a dual reporter transgenic mouse line (*ROSA26*^*tm4.ACTB-tdTomato-EGFP*^), which allows us to capture cell morphogenesis and migration of somatic and CNCCs. The illustrative diagram depicts the procedure for dissecting the neural folds from embryos at E8.5 (Fig. 4A). We used this system to uncover the function of *Twist1* in pre-EMT CNCCs, wherein lack of its expression in the neuroectodermal and pre-EMT neural crest cells can explain the phenotype observed in *Twist1*^*cko/*−^. The cells expressing the *Wnt1-Cre* transgene excise the membrane-associated *mTomato* reporter gene, allowing instead the expression of *GFP*. In wild-type explants, individual migratory CNCCs moved away from the dorsolateral sides of the neural folds (Fig. 4B,B′) and the migratory mesenchymal cells formed long protrusions (inset in Fig. 4B′). In the *Twist1*^*cko/*−^ explant, a considerable amount of *Twist1*-deficient neuroectodermal cells remained within the neural folds and expanded ventrally (Fig. 4C), and the majority of the detached CNCCs did not transition into mesenchymal cells but instead maintained their cell-cell adhesion and epithelial morphology (Fig. 4C′, inset in C′). Quantitative measurements of the total number of migratory CNCCs were analyzed for each genotype. We detected approximately a 50% reduction in the total number of cells in mutant explants (Fig. 4D), and the average distance of all migratory CNCCs was reduced by 68% (Fig. 4E). We also measured the mean speed of individual CNCCs in wild type and *Twist1*^*cko/*−^. To determine the mean speed of individual CNCCs, we tracked the CNCCs as they moved away from the neural tube in wild type and *Twist1*^*cko/*−^ (Fig. 4F,F′,G,G′). Micrographs show individual CNCC migration paths during time-lapse imaging (Fig. 4F′,G′, white lines). Wild-type CNCCs mean speed increased by 50% in comparison with *Twist1*^*cko/*−^ (Fig. 4H).

### Neural tube phenotype in *Irf6* null embryos

**Fig. 5:**
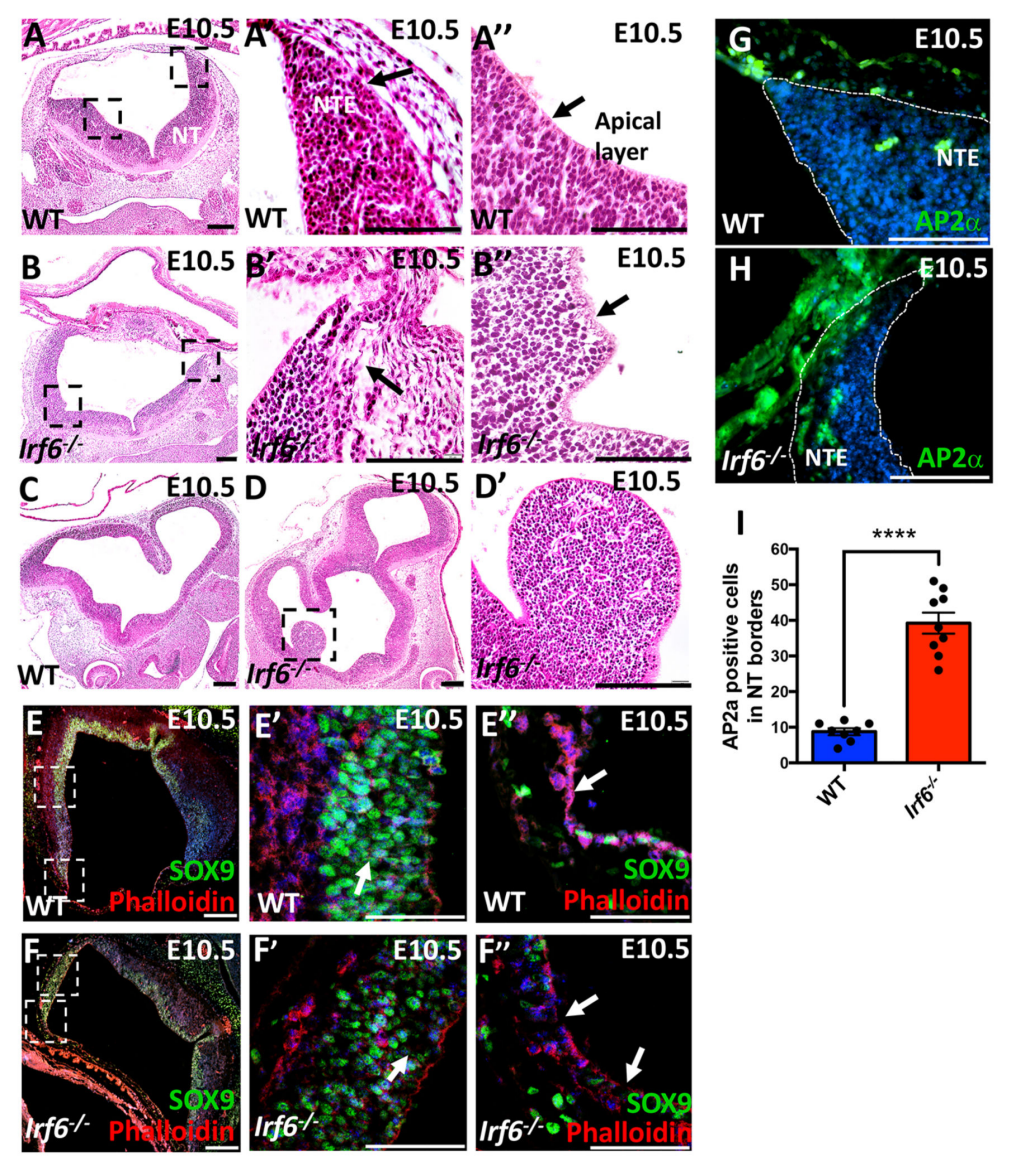
Neural tube phenotype in *Irf6*-null embryos. (A-A″) Hemotoxylin and Eosin staining of wild-type sections shows the morphology of the NT (A), the NT dorsal edges (NTE) (A′) and NT apical cells (A″) at E10.5. (B-B″) Hemotoxylin and Eosin staining of *Irf6*-null sections shows the NT morphology (B), NT edge with less organized neuroepithelial cells and larger intercellular space (B′), and NT apical layer showing a wavy pattern (B″). Hemotoxylin and Eosin staining of wild-type section shows the forebrain morphology. (D,D′) Abnormal protrusions from the brain cortex are detected in *Irf6*-null embryos. (E-F″) Expression of SOX9 and F-actin was detected in wild-type (E-E″) and *Irf6*-null embryos (F-F″). (E′,F′) The organization of SOX9-positive neuroepithelial cells is altered, with increased intercellular space between the cells in *Irf6*-null embryos (F′) compared with wild type (E′). (F,F″) The integrity of the apical membrane of NT is altered in *Irf6* null embryonic section (F″) compared with wild type (E″). The top and bottom squares in E and F are shown at higher magnification in E′,F′ and E″,F″, respectively. (G) Immunostaining of AP2α in green shows expression in ectoderm and the junction between non-neural and NT edge marked with dotted line in wild type. (H) Increased expression AP2α is detected in the NT edges and the junction between non-neural ectoderm and NT edge. (I) The quantification of the AP2α-positive cells at the NT edges shows a significant increase in *Irf6*-null embryos compared with wild-type littermates. Data are mean±standard error of the sampling distribution. ****P<0.0001. Scale bars: 100 μm.

IRF6 is expressed in the non-neural and neural ectoderm during neural tube formation. Its expression is relatively stronger in neural ectoderm than non-neural ectoderm (Fig. 1E,E′; Fig. S1C-E). We hypothesized that a complete loss of *Irf6* affects neural tube formation, leading to craniofacial abnormalities. At E10.5, we noticed that the cellular organization of the edges of neural tube and apical membrane were disorganized in *Irf6* null compared with wild-type littermates (Fig. 5A-B″, black arrow). Abnormal expansions from the brain cortex were also detected in *Irf6-*null embryos compared with wild type (Fig. 5C-D′). We checked the expression of SOX9, which is expressed in the neuroepithelial progenitors of glia (Farrell et al., 2011; Vogel and Wegner, 2021), and F-actin in wild-type and *Irf6*-null embryos. SOX9-positive cells were detected in the dorsal half of the neural tube (Fig. 5E,E′, white arrow). However, we found that the organization of SOX9-positive cells was altered and large intercellular gaps among SOX9 positive cells were detected in *Irf6*-null neural tube (Fig. 5F,F′, white arrows). A continuous expression of F-actin was noticed in the apical cells of the dorsal edge of wild-type neural tube (Fig. 5E″), while the apical membrane was discontinuous in *Irf6*-null embryos (Fig. 5F″). We also investigated the expression of non-neural ectodermal marker, AP2α, to uncover whether a loss of *Irf6* alters its expression at the neural tube junction. We noticed that the number of AP2α-positive cells at the neural tube dorsal edges increased in *Irf6*-null embryos compared with wild-type littermates in four different biological replicates of each genotype (Fig. 5G,H). We quantified the number of AP2α-positive cells in the dorsal edges and observed a significant increase in *Irf6* null compared with wild type (Fig. 5I).

### Expression pattern and level of epithelial genes and SOX9 in neural tube and migratory mesenchymal cells

**Fig. 6:**
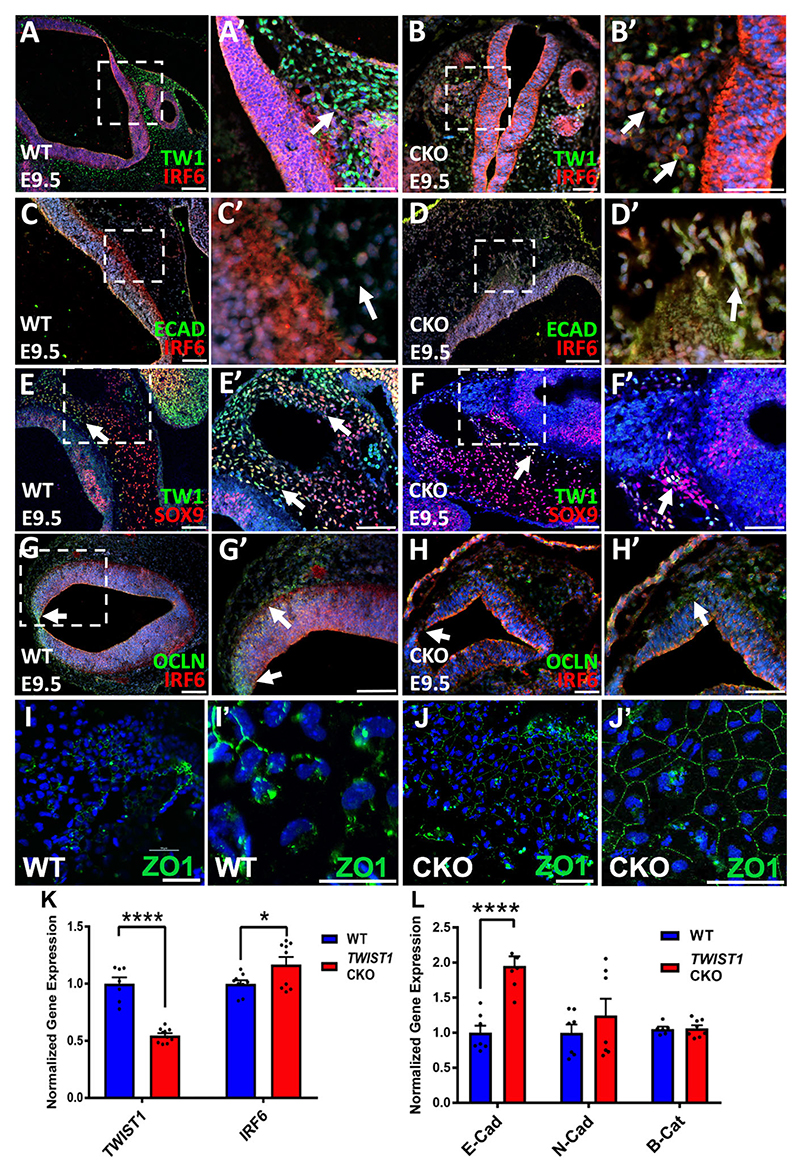
Expression of IRF6, TWIST1 and other factors during NT and CNCC formation. (A,A′) IRF6 is detected in NT and TWIST1 is expressed in migratory CNCCs at E9.5. (B,B′) IRF6 expression in *Twist1*^*cko/*−^ embryos is detected in the partially delaminated CNCCs along the NT. (C,C′) Staining for E-cadherin in wild type shows expression in ectoderm and slightly at apical regions of NT. (D,D′) In *Twist1*^*cko/*−^ embryos, ectopic E-cadherin expression is detected in the partially detached and migratory mesenchymal cells. (E,E′) TWIST1 and SOX9 co-expression in yellow-green is observed in migratory CNCCs in wild type (white arrows). (F,F′) Few CNCCs co-express TWIST1 and SOX9 in *Twist1*^*cko/*−^. (G-H′) Occludin is highly expressed at the junction of the NT edges (G, G′), whereas its expression is remarkably reduced in *Twist1*^*cko/*−^ (H,H′). Arrows in A′,B′,C’,D′,E-F′ indicate expression of marker genes in mesenchymal cells. (I-J′) ZO1 is detected as an interspaced and broken signal in wild-type CNCCs (I,I′), whereas its expression is continuous at the junction between the detached CNCCs of *Twist1*^*cko/*−^ explants (J,J′). (K) *Irf6* mRNA expression shows a slight increase in the *Twist1*^*cko/*−^. Squares in A,B,C,D,E,F,G indicate the areas shown at higher magnification in A′,B′,C′,D′,E′,F′,G′. (L) mRNA of E-cadherin, N-cadherin and β-catenin were measured in wild type and *Twist1*^*cko/*−^. E-cadherin expression is significantly increased in *Twist1*^*cko/*−^. *P<0.05, ****P<0.0001. Data are mean±standard error of the sampling distribution. Scale bars: 50 μm.

Based on the CNCC-derived tissue phenotypes observed in *Twist1*^*cko/*−^, we investigated the expression pattern and level of SOX9 and epithelial genes involved in cell adhesion and differentiation to determine why a loss of *Twist1* in delaminated CNCCs maintained their epithelial morphology. In wild-type embryos, IRF6 was mainly detected in the neural tube, whereas TWIST1 was highly expressed in migratory mesenchymal cells that were detached from the neural tube and migrated towards frontonasal and pharyngeal prominences at E9.5-E10.5 (Fig. 6A,A′; Fig. S7A-C). Ectopic IRF6 expression in partially detached mesenchymal cells was detected in a cluster of cells along the neural tube in *Twist1*^*cko/*−^ embryos (Fig. 6B-B′, white arrow). No expression of E-cadherin (E-CAD) was present in mesenchymal cells in wild type (Fig. 6C,C′). However, staining for E-CAD in *Twist1*^*cko/*−^ embryos showed that the partially detached mesenchymal cells express ectopic E-CAD (Fig. 6D,D′). SOX9 expression was used to mark migratory CNCCs along the neural tube and to distinguish CNCCs from mesodermal cells. In a wild-type embryo, an abundant number of migratory CNCCs showed co-expression of SOX9 and TWIST1 (Fig. 6E,E′ white arrows), whereas only a few TWIST1-positive CNCCs can be seen in the *Twist1*^*cko/*−^ embryo (Fig. 6F-F′, white arrow). We also looked at the expression of tight junction proteins occludin and ZO1. Occludin is expressed at high levels at the dorsal junction of neural tube edges of wild-type embryos (Fig. 6G,G′), whereas almost no expression of was detected at the unfused junction of the dorsal edges of *Twist1*^*cko/*−^ neural tube (Fig. 6H,H′). In neural tube explants, an interspaced and broken signal of ZO1 was seen among the migratory mesenchymal cells in wild type (Fig. 6I,I′), whereas a continuous junctional signal of ZO1 was observed among detached mesenchymal cells from the neural tube of *Twist1*^*cko/*−^ (Fig. 6J,J′). We also looked at the expression of a mesenchymal marker, vimentin, and observed a disrupted expression pattern in the mesenchymal cells in *Twist1*^*cko/*−^ embryos and neural tube explants compared with wild type (Fig. S7D-G′). As expected, *Twist1* expression was significantly reduced in *Twist1*^*cko/*−^ embryos, and a slight increase was seen in *Irf6* mRNA in *Twist1*^*cko/*−^ (Fig. 6K). mRNA encoding *E-Cad* (*Cdh1*) *N-Cad* (*Cdh2*) and β-catenin (*Ctnnb1*) were also measured in WT and *Twist1*^*cko/*−^ (Fig. 6L). *E-Cad* expression was significantly increased in *Twist1*^*cko/*−^ (Fig. 6L), which is consistent with the ectopic expression of E-CAD in partially detached mesenchymal cells (Fig. 6D,D′).

### Quantitative levels of kinases, signaling molecules, and epithelial and mesenchymal marker genes

The expression of genes encoding kinases [*Akt1, Akt2*, members of the Pi3k family, *Erk1* (*Mapk3*), members of the Ck2 family and *Src*]; epithelial markers [*E-Cad, N-Cad* and occludin (*Ocln*)]; neural and non-neural ectoderm markers, and CNCC specification drivers [*Snail2* (*Snai2*), *Msx1, Tfap2a, Hand2, Sox2, Sox10* and *Hoxd10*]; signaling molecules [*Yap* (*Yap1*), *Wnt1, Wnt3, Jag1* and *Mtor*]; and actomyosin remodeling regulators (β-catenin, *Rhoa, Rhoc* and *Arhgap29*) was measured in dissected tissues of the hindbrain and first pharyngeal arch (Fig. S8). The mRNA expression of *Akt2, Snai2, Msx1, Hand2, Sox10, Yap*, β-catenin, *Rhoa* and *Arhgap29* was significantly reduced in *Twist1*^*cko/*−^ embryos at E10.5 (Fig. S8A,E,I,G,K). Protein analysis by western blot also showed decreased levels of AKT1, AKT2, AKT3, SNAI2 and WNT3 in *Twist1*^*cko/*−^ embryos (Fig. S8B,F,H). Further details are explained in the legend of Fig. S8.

### TWIST1 nuclear expression and phosphorylation *in vivo* and in the O9-1 cell line

TWIST1 has six phospho-residues that are highly conserved in vertebrates (Fig. S9A). Our data show that some subpopulations in the dorsal edges of neural folds and in the pre-EMT CNCCs express TWIST1 mostly in the cytosol (Fig. S9B-C′, arrows; Fig. S1A′,D, D′), and it becomes mainly nuclear in detached and migratory CNCCs at E9.5 and E10.5 (Fig. 6A,A′, arrow; Fig. S9D,D′; Fig. S7C). Our *in vivo* data show that phosphorylated TWIST1 protein can be detected in mesenchymal cells using monoclonal antibodies raised against TWIST1 phospho-S68, and polyclonal antibodies raised against TWIST1 phospho-S42 and phospho-S123 residues at E10.5 (Fig. S9E). TWIST1 unphosphorylated and phosphorylated forms were also detected in the O9-1 cell line (Fig. S9F). Further details are provided in the legend of Fig. S9.

### Importance of TWIST1 phosphorylation in craniofacial tissues

**Fig. 7:**
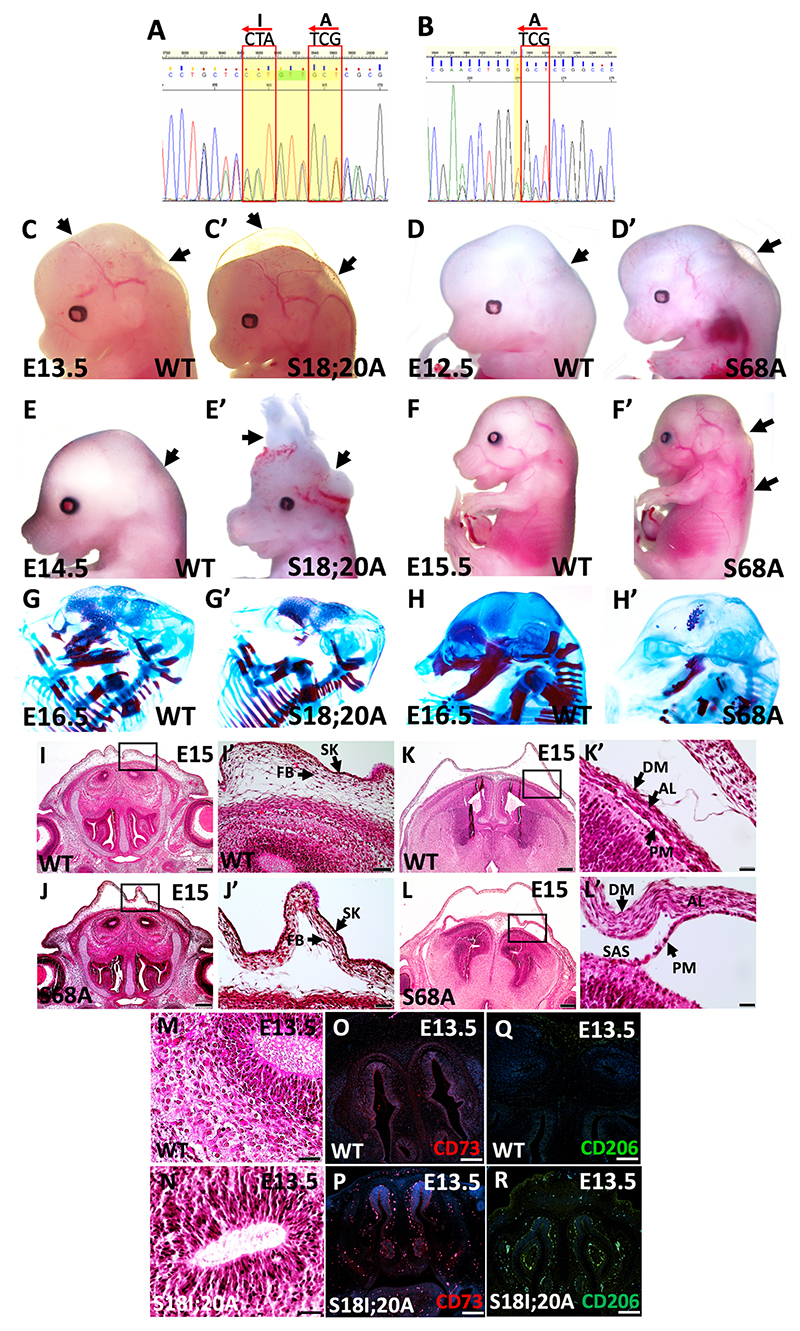
The *Twist1*^*S18I;20A/S18I;20A*^ and *Twist1*^*S68A/S68A*^ morphological and skeletal phenotype. (A) The DNA sequences show the changes in the serine residue S18/20 to isoleucine and alanine, and S68 to alanine. (C-F′) *Twist1*^*S18I;20A/S18I;20A*^ (C′,E′) and *Twist1*^*S68A/S68A*^ (D′,F′) mice show epidermal blebbing, severe edema along the neural tube and neural tube defects. Arrows indicate areas of normal development in wild-type embryos versus severe edema and malformation in mutant embryos. (G-H′) Alizarin Red and Alcian Blue skeletal staining. (G,G′) The *Twist1*^*S18I;20A/S18I;20A*^ mutant embryo has no mandibular and maxillary bone compared with wild type. (H,H′) *Twist1*^*S68A/S68A*^ shows a remarkable reduction in skull mineralization of the frontonasal and maxillary bone compared with wild type at E16.5. (I-L′) Hemotoxylin and Eosin staining. (I-K′) Normal morphology of frontonasal structures of a coronal section in wild type. (J-L′) The *Twist1*^*S68A/S68A*^ embryo has subepidermal blebbing, detachment of fibroblast layers from brain and meninges abnormalities compared with wild type. (M,N) Hemotoxylin and Eosin staining in the brain of *Twist1*^*S18I;20A/S18I;20A*^ shows infiltration of lymphocytes compared with wild type. (O-R) Immunofluorescence staining shows no signal for CD73 and CD206 in wild type (O,Q); however, strong staining was observed in the brain and around nasal cavities of *Twist1*^*S18I;20A/S18I;20A*^ (Q,R). Scale bars: 100 μm.

To determine the importance of TWIST1 post-translational modifications in the neural tube and CNCC-derived tissues, we generated four phospho-incompetent mouse lines, including *Twist1*^*S18;20A*^, *Twist1*^*S42A*^, *Twist1*^*S68A*^ and *Twist1*^*T121A;* *S123A*^ using CRISPR/CAS9 technology. The founders of *Twist1*^*S42A*^ mice did not give any progeny, and all ten *Twist1*^*T121A;* *S123A*^ founders died before reaching 2 months of maturity. We confirmed the genomic changes in three founders of *Twist1*^*S18I;20A/+*^ and in four founders of *Twist1*^*S68A/+*^. The sequence of F6 generations confirmed the changes of serine 18 (S18) to isoleucine and serine 20 (S20) to alanine, and in the second mouse line, serine 68 (S68) to alanine (Fig. 7A,B).

The phenotypic characterization showed that the two phosphorylation sites, S18/20 and S68, are crucial for TWIST1 activity in craniofacial development. The two phospho-incompetent mouse lines have epidermal blebbing, severe edema along the neural tube and significant neural tube defects in *Twist1*^*S18I;20A/S18I;20A*^ compared with wild-type littermates (Fig. 7C-F′). The skeletal staining exhibited a remarkable loss of maxillary and mandibular bones in *Twist1*^*S18I;20A/S18I;20A*^ and a reduction in skull mineralization in *Twist1*^*S68A/S68A*^, respectively (Fig. 7G′,H′), compared with wild type (Fig. 7G,H). The histological staining of *Twist1*^*S68A/S68A*^ head structures at E15 showed subepidermal blebbing, separation of fibroblast layers from the meninges and a detachment of the meninges from the surface of the brain cortex (Fig. 7J,J′,L,L) compared with wild-type littermates (Fig. 7I,I′,K,K′). We also noticed lymphocyte infiltration in *Twist1*^*S18I;20A/S18I;20A*^ brain tissues and around the frontonasal processes compared with wild-type embryos (Fig. 7M,N). To determine the types of the infiltrated cells, immunofluorescence staining for the lymphocyte marker CD73 and B cell marker CD206 was used on tissues sections at E13.5. No signal was detected in the frontonasal sections of wild type (Fig. 7O,Q); however, enrichment of both markers was noticed in the frontonasal tissues of *Twist1*^*S18I;20A/S18I;20A*^ embryos (Fig. 7P,R). The genotype and spectrum of craniofacial phenotype in the homozygous phospho-incompetent embryos are further described in Fig. S10.

### Interaction between *Twist1* and *Specc1l* in CNCCs

**Fig. 8:**
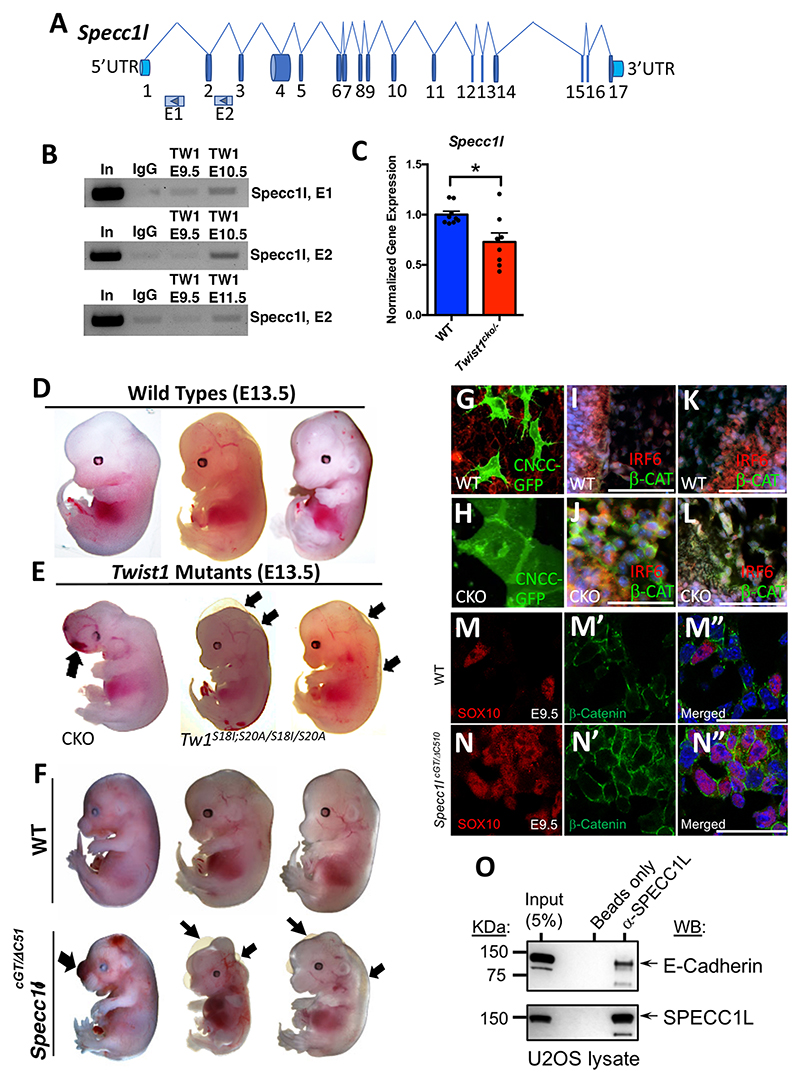
TWIST1 regulates *Specc1l* expression. (A) Schematic of the *Specc1l* gene. (B) TWIST1 binds to two *Specc1l* putative enhancer elements within intron 1 (E1) and 2 (E2) when assessed in dissected tissues by ChIP-PCR. (C) *Specc1l* is significantly decreased in *Twist1*^*cko/*−^ embryos compared with wild type. (D) Wild-type embryos show normal craniofacial development. (E) *Twist1*^*cko/*−^ and *Twist1*^*S18I;20A/S18I;20A*^ mutant embryos display craniofacial hemorrhage, subepidermal blebbing and edema along the NT at E13.5. Arrows indicate affected regions. (F) Images of wild-type embryos in comparison with *Specc1l*^*cGT/DC510*^ mutant embryos. The *Specc1l*^*cGT/DC510*^ mutant embryos show frontonasal hemorrhage, subepidermal blebbing and edema along NT. Arrows indicate affected regions. (G,H) Confocal images show individual migratory wild-type CNCCs and a cluster of epithelial-like cells in the *Twist1*^*cko/*−^ explant. (I,J) Immunofluorescence images show no expression of β-catenin in mesenchymal cells of wild type and a pronounced expression in partially detached mesenchymal cells in *Twist1*^*cko/*−^. (K,L) No expression of E-cadherin in mesenchymal cells of wild type; a robust signal in partially detached mesenchymal was observed in *Twist1*^*cko/*−^. (M-M″) Immunofluorescence images show SOX10 expression and scattered expression of β-catenin in migratory CNCCs of wild type. (N-N″) Immunofluorescence images show SOX10 expression and pronounced membrane-associated expression of β-catenin in migratory CNCCs of *Specc1l*^*cGT/DC510*^ embryos. (O) A co-IP blot in U2OS cells shows the E-cadherin is pulled down with SPECC1L protein, as shown in the western blot after immunoprecipitation with anti-SPECC1L antibodies. *P<0.05. Data are mean±standard error of the sampling distribution. Scale bars: 50 μm.

Many of the tested cytoskeletal remodeling regulators were significantly reduced at the mRNA level in *Twist1*^*cko/*−^ embryos. We focused on *Specc1l* because of the phenotypic overlap in craniofacial disorders in humans and mice, and the importance of this gene in regulating the actomyosin cytoskeleton. We tested the hypothesis that TWIST1 regulates *Specc1l* expression in CNCCs and that both factors interact with adherens junction proteins. *Specc1l* has 17 exons plus 5′ and 3′ UTR (Fig. 8A). We mapped two putative regulatory elements in intron 1 and 2 based on epigenetic signatures (Fig. S11). We performed ChIP-PCR to determine whether TWIST1 *in vivo* binds to E1 and E2 putative elements. The results showed enrichment of TWIST1 protein at the putative *Specc1l* element E1 for both embryonic time points E9.5 and E10.5. The bound TWIST1 to E2 putative enhancer was increased at E10.5 as indicated by the PCR band intensity but not at E9.5 or E11.5 (Fig. 8B). mRNA expression of *Specc1l* was significantly reduced in tissues extracted from the hindbrain and first pharyngeal arch of *Twist1*^*cko/*−^ compared with wild type (Fig. 8C). We matched the phenotype in *Twist1*^*cko/*−^ and phospho-incompetent embryos with *Specc1l* loss-of-function mutant mouse embryos to compare the phenotypic overlap in craniofacial tissues and CNCCs. *Twist1*^*cko/*−^ and phospho-incompetent mutant embryos showed brain hemorrhage, subepidermal blebbing and severe edema along the neural tube (Fig. 8D,E). Similarly, the *Specc1l*^*cGT/DC510*^ compound mutant alleles (described by Hall et al., 2020) show brain hemorrhage, subepidermal blebbing and severe edema along the neural tube compared with wild-type littermate embryos (Fig. 8F). Loss of *Twist1* in CNCCs led to disruption in the EMT process and the persistence of cell-cell adhesion in migratory mesenchymal cells (Fig. 8H), and the *in vivo* accumulation of adherens junction proteins β-catenin and E-CAD in partially detached cells (Fig. 8J,L; Fig. S12B-B‴ and D-D‴) compared with wild type (Fig. 8G,I,K; Fig. S12A-A‴,C-C‴). Similarly, loss of *Specc1l* function caused the accumulation of membrane-associated β-catenin in migratory CNCCs (Fig. 8N-N″) compared with wild-type CNCCs (Fig. 8M-M″). We tested whether SPECC1L protein interacts with adherens junction proteins using U2OS cell lysate due to high expression in this cell line. SPECC1L and E-CAD were detected in the input of total protein lysate. E-CAD protein was pulled down with SPECC1L in a co-IP assay in a lysate immunoprecipitated by anti-SPECC1L antibodies, and no band was detected in the negative control (Fig. 8O).

### Common variants near *TWIST1* impact human facial shape

**Fig. 9:**
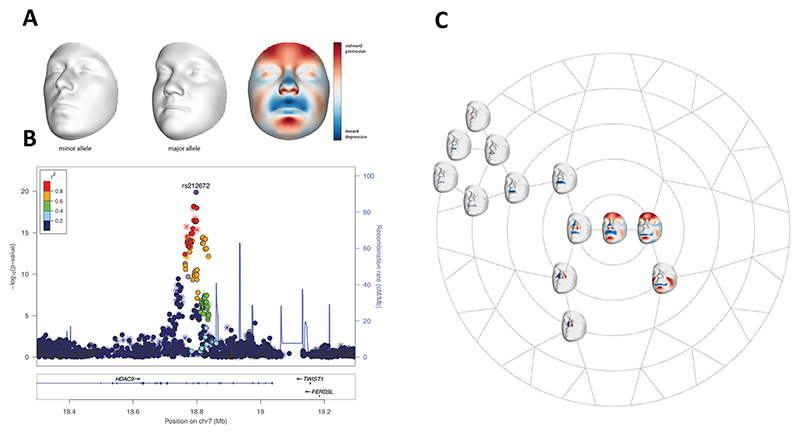
GWAS of 3D human facial surfaces showing a strong signal near the *TWIST1* locus. (A) The 3D facial surfaces show the phenotypic effects of the lead SNP on the whole face, exaggerated in the direction of the minor (A) and major (T) allele SNP variant. (B) The points in the LocusZoom plot are color coded based on linkage disequilibrium (*r*^2^) in Europeans with lead SNP rs212672. The asterisks represent genotyped SNPs; the circles represent imputed SNPs. (C) The branching rosette diagram shows the effect of the lead SNP on various facial segments, following unsupervised hierarchical clustering of the full facial surface into increasingly smaller subparts. The nodes in this diagram represented by 3D facial surfaces are the facial segments where the lead SNP reached the nominal genome-wide *P*-value threshold (*P*=5×10^−8^). The heatmaps on each 3D surface show the anatomical boundaries of the segments. The smallest *P*-value was observed on the whole face. The other facial segments impacted by the lead SNP involved the nose and upper lip.

The different mouse models of *Twist1* loss of expression and function indicate the crucial role of this gene in regulating the development of CNCC-derived structures. To determine the relevance of these animal model findings to humans, we revisited a recently published GWAS meta-analysis of 8246 individuals of recent European ancestry, which identified 203 genome-wide significant signals associated with normal-range facial shape, as previously described by White et al. (2021). When moving from the minor (A) to major (T) allele of the leading SNP, the major shape changes include a less protrusive and wider nose, a shorter and less protrusive upper lip, and a more prominent chin and forehead. These changes are visible in the morphs and in a heatmap that shows the same effect using the normal displacement at each point comprising the facial surface (~8000 points), with blue representing inward depression and red representing outward protrusion (Fig. 9A). A signal at 7p21.1 near *TWIST1* was significantly associated facial shape and the lead SNP (rs212672) is located ~355 kb downstream of *TWIST1* (Fig. 9B). When the face was partitioned into global-to-local anatomical segments, the strongest association with the lead SNP was observed for the whole face (*P*=1.31E^−20^) and for CNCC-derived facial regions involving the nose and upper lip (see rosette diagram in Fig. 9C).

## Discussion

Uncovering the underlying mechanism of *Twist1* and *Irf6* function in cell fate regulation is crucial to identify their regulatory pathways and associated genes that can control tissue development and differentiation. Our work tested the hypothesis that *Twist1* is involved in neural tube formation and EMT of CNCCs, while *Irf6* helps define neural tube dorsal edges and structural integrity. Our immunofluorescence staining data showed that TWIST1 expression has a similar pattern to β/δ-catenin and tight junction proteins at the apical side of neural plate and neural folds. This cellular overlap is validated by the dual immunofluorescence of TWIST1 and β-catenin. Furthermore, the *in situ* hybridization results showed that *Twist1* mRNA is also weakly expressed in the dorsal edges of neural plate and apical side of neural folds. Our findings are consistent with a recent study indicating that a few cells of the neural folds express *Twist1* mRNA when detected using single cell RNA-seq technology (Soldatov et al., 2019), although the scRNA-seq data relies on computational analysis to determine the spatial expression and the relative anatomical distribution of this population of cells (Soldatov et al., 2019). Additionally, *Twist1* expression was detected in round cells residing in the neuroectoderm of the forebrain during the emergence of CNCCs at E8.5 (Füchtbauer, 1995).

To provide valuable insight on TWIST1 apical expression in the neural folds, we explored whether TWIST1 is co-expressed with LRP2 and RAB11b in endocytic vesicle compartments. We based our investigation on a previous report on LRP2 and RAB11b co-expression in endocytic compartments at the apical surface of neural folds (Kowalczyk et al., 2021). We found that TWIST1 is expressed in endocytic vesicles at the apical surface of neural folds, and its expression partially overlaps with RAB11b and LRP2. Recycling endosomes are important for apical constriction, and loss of *Lrp2* leads to neural tube closure defects in mouse embryos (Kowalczyk et al., 2021). Similarly, loss of *Twist1*, β-catenin and δ-catenin in neuroectodermal cells leads to neural tube closure defects (Kowalczyk et al., 2021; Brault et al., 2001; Pieters et al., 2020). The cellular fractionation co-IP data shows that the cytosolic TWIST1 interacts with β/δ-catenins during neural tube closure, and *Twist1* CKO using *Wnt1-Cre* disrupts the apical expression of β-catenin, which mostly becomes cytosolic. Notably, δ-catenin CKO by *Wnt1-Cre* also causes a focal loss of N-cadherin at the apical side of neural folds (Pieters et al., 2020). However, we cannot definitively disentangle the cytosolic versus nuclear contribution of *Twist1* because it is a dynamic and continuous process, and the disruption of the neural tube formation might impact the delamination of the CNCCs. Therefore, our data suggests that the interaction of cytosolic TWIST1 with β/δ-catenins, and the expression of TWIST1 in endocytic vesicles at the apical surface might facilitate the apical constriction of neuroectodermal cells, whereas lack of *Twist1* in neuroectodermal cells leads to an altered cell shape with multiple ectopic dorsolateral hinge points at the apical surface of the neural tube. Our findings are consistent with a recent publication showing that TWIST1 interacts with tight junction protein 1 (TJP1) along the cell membrane of two different cancer cells (Liu et al., 2022). Liu et al. also demonstrated that TWIST1 expression overlaps with TJP1 in vesicles at cellular membrane (Liu et al., 2022).

*Twist1*-null mice exhibited degeneration of the apical neuroectodermal cells, and increased cell death in migratory CNCCs (Bildsoe et al., 2009; Chen and Behringer, 1995; Soo et al., 2002). Although the adjacent cephalic mesenchyme activity contributes to neural fold elevation, it does not explain why the neural folds in *Twist1*-null embryos do elevate but fail to bend and close completely. Similarly, in *Twist1*^*cko/*−^ in CNCCs, the neural folds bend as if to join but stop prematurely, leading to improper closure of the neural tube. Although the phenotypes caused by a lack of *Twist1* in CNCCs and mesoderm can be explained by an indirect role of *Twist1* in regulating neural tube formation (Bildsoe et al., 2013), *Twist1* expression in the neural folds provides a complementary mechanism for explaining the neural tube closure defects in *Twist1*-null embryos, the multiple ectopic bending points and the expansion in the neural tube of *Twist1*^*cko/*−^. Although a complete loss of *Twist1* did not disrupt the anterior-posterior patterning of the neural tube at E9.5, there was expansion in a few components of Shh and Fgf pathways (Soo et al., 2002). A direct role in the expansion of these components has not been previously reported because *Twist1* expression was not described in the neural folds (Soo et al., 2002). *Twist1* CKO in mesodermal cells disrupts neural fold elevation (Bildsoe et al., 2013), and the rescue experiment with wild-type mesenchymal cells in *Twist1*-null chimera embryos indicated that cephalic mesenchymal cells are involved in neural fold elevation (Chen and Behringer, 1995). For the function of IRF6 in neural tube, we have shown that overexpression of *Irf6* in the basal layer of non-neural ectoderm suppresses *Tfap2a* in the ectoderm and causes neural tube abnormalities and exencephaly (Kousa et al., 2019). Our current findings show that AP2α expression was expanded into the neural tube in *Irf6*-null embryos, which could explain the disruption of neural tube edge integrity. In addition, the glial progenitor cells within the neural tube were disorganized in *Irf6*-null embryos with more noticeable gaps between the progenitor cells.

The *Wnt1* ligand is a crucial regulator in the identification of neural plate border domains and in early induction of CNCCs (Parr et al., 1993). The *Wnt1-Cre* transgene drives the expression of *Cre* throughout the neural plate at E8.5 and later stages during neural tube development (Chang et al., 2015). Hence, *Wnt1-Cre* was ideal to delete *Twist1* at early stages, if present, during early neural plate formation. Previous studies have suggested that *Twist1* CKO in neuroectoderm by *Wnt1-Cre* does not disrupt CNCC migration towards the frontonasal and pharyngeal processes. However, the X-gal staining in *Twist1*-deficient CNCCs was reduced in frontonasal and pharyngeal arches, as *Twist1* was considered a survival factor in migratory CNCCs (Chen et al., 2007, 2017; Zhang et al., 2012). Apoptosis in *Twist1*-deficient CNCCs might be one of the disrupted cellular functions behind the loss of craniofacial bone and cartilage. Yet no report has indicated whether mammalian *Twist1* is involved in the events before CNCC delamination or whether the migratory *Twist1*-deficient CNCCs are normal mesenchymal cells. The data in this study from neural tube explants of embryos shows that *Twist1* regulates EMT in CNCCs and that a loss of *Twist1* in neuroectoderm leads to disruption of cell fate transition and delamination. Our findings also show that TWIST1 suppresses the expression of *Irf6* and other epithelial factors during delamination. *Irf6* is a terminal differentiation factor of proliferative epithelial cells in the ectoderm and its expression prevents EMT (Fakhouri et al., 2017; Ingraham et al., 2006; Thompson et al., 2019). In agreement with its role in EMT, *Twist1* overexpression in the trunk neural tube converts the pre-EMT neuroectodermal cells into cells that resemble CNCCs (Soldatov et al., 2019). Recent studies on the EMT show that a complete conversion from an epithelial to a mesenchymal state is not necessary for cancer cells to migrate and multiple metastable states during cell transition have been described in cancer studies (Cousin, 2017; Nieto et al., 2016; Thiery et al., 2009). However, we did not observed a collective cell migration or epithelial-like cells in wild-type explants or *in vivo* histological staining. Lineage analysis may be the most appropriate way to detect the effect of *Twist1* loss on target genes. Unfortunately, the lack of such analysis limits our interpretation of the impact of *Twist1* on target gene expression in migratory CNCCs.

Determining the *in vivo* role of TWIST1 post-translational modification is vital in delineating its role in neural tube and CNCCs. TWIST1 is a phospho-protein and multiple phospho-residues are necessary for its stability, nuclear translocation and transcriptional regulation, as per previous cancer studies (Bourguignon et al., 2010; Zhao et al., 2017; Xu et al., 2017). *Twist1* phosphorylation increases tumor cell motility in squamous cell carcinoma of the head and neck (Alexander et al., 2006; Su et al., 2011). In addition, overexpression of *Twist1* phosphomimetic T125D; S127D led to limb abnormalities in transient transgenic mouse embryos (Firulli et al., 2007). We suggest that post-translational phosphorylation of TWIST1 is crucial for regulating its cellular activity in CNCCs. The *Twist1*^*S18;20A/S18;20A*^ and *Twist1*^*S68A/S68A*^ mutants embryos showed several craniofacial abnormalities, including epidermal blebbing, severe edema, meningeal detachment and neural tube defects. The skeletal staining of these phospho-incompetent mutant embryos showed bone loss and reduced skull mineralization of the frontonasal and maxillary bone. Yet the *in vivo* molecular function of the two phospho-sites needs to be delineated, including its importance in protein stability, nuclear translocation, the EMT process, cell survival and differentiation.

The data generated on the differentially expressed genes in *Twist1*^*cko/*−^ embryos demonstrated that many factors involved in intercellular adhesion proteins and cytoskeletal remodeling are significantly altered. Previous studies have shown that mutations in adhesion protein and cytoskeletal regulator genes cause neural tube closure defects and improper delamination of CNCCs, leading to craniofacial disorders (Pieters et al., 2020; Wilson et al., 2016). One of the genes contributing to this delamination defect in mice is *Specc1l*. Mutations in TWIST1 and SPECC1L lead to craniosynostosis and orofacial clefting in humans (Bai et al., 2021; Bhoj et al., 2019; Goering et al., 2021; Hall et al., 2020). *Specc1l* is a gene that encodes a novel coiled-coil domain-containing protein, which stabilizes microtubules and actin of the cytoskeleton (Bhoj et al., 2019; Gfrerer et al., 2014). SPECC1L colocalizes with both cellular actin and tubulin (Saadi et al., 2011; Wilson et al., 2016). Thus, without sufficient SPECC1L, actin-cytoskeleton reorganization and cell adhesion are significantly impacted (Saadi et al., 2011). The overlap in cellular function and embryonic phenotype between *Twist1* and *Specc1l* mutant mouse lines suggests that both genes are involved in a similar regulatory pathway to control cytoskeleton reorganization during cell delamination and migration. This study shows that TWIST1 directly binds to putative regulatory elements in the intronic regions of *Specc1l* and that loss of *Twist1* leads to a significant reduction of *Specc1l* in hindbrain and first pharyngeal arch tissues, suggesting that TWIST1 acts as an activator for *Specc1l*.

In conclusion, our findings emphasize the direct role of Twist1 and Irf6 in neural tube development, and the conservation of TWIST1 function in regulating the EMT process in CNCCs by controlling the expression of epithelial genes and cytoskeletal regulators. *Specc1l* and *Twist1* play a similar role in the delamination of CNCCs by remodeling the cell-cell adhesion and cytoskeletal reorganization. Thus, previous reports and the current study provide a reasonable explanation for the overlapping phenotypes in mice and humans. In addition, as our results highlight, reanalysis of the GWAS data in humans shows that variants near *TWIST1* impact normal-range facial shape, including CNCC-derived structures of the midface (Claes et al., 2018; White et al., 2021). Notably, the most significant SNP near *TWIST1* overlaps with epigenetic enhancer signatures in which the elements with the epigenetic marks were tested for enhancer activities to recapitulate TWIST1 endogenous expression (Hirsch et al., 2018). Understanding the transcriptional regulation of *TWIST1* and its function in controlling the associated regulatory pathway involved in cell fate determination could lead to the identification of the missing heritability in families that are affected and at high risk of craniofacial birth defects.

## Materials And Methods

### Mouse strains

All the mice used in this study were generated using the C57BL/6J genetic background. *Twist1* heterozygous mice were generated using *EIIA-Cre* mouse line using the B6;129S7-*Twist1*^*fl/fl*^ [obtained from the Mutant Mouse Resource and Research Center (MMRRC) supported by the National Institutes of Health). The mouse lines 129S4.Cg-*E2f1*^*Tg(Wnt1-cre2)Sor*^/J (022137), BL6.129(Cg)-*Gt(ROSA)26Sor*^*tm4(ACTB-tdTomato*,−*EGFP)Luo*^/J (007676) and BL6.129S4-*Gt(ROSA)26Sor*^*tm1Sor*^/J (003474) were obtained from the Jackson Laboratory. The two different cell-tracing strategies R26^*tm4(ACTB-tdTomato*,−*EGFP)*^ and R26^*tm1.lacZ*^ were used to track *in vivo* CNCC formation and migration. *Twist1*^*S18;20A/+*^, *Twist1*^*S42A/+*^, *Twist1*^*S68A/+*^ and *Twist1*^*T121;S123A/+*^ phospho-incompetent founders were generated using CRISPR/Cas9 method at the Baylor College of Medicine. We backcrossed all phospho-mutant founders to C57BL/6J wild-type mice for six generations to avoid non-specific genomic alterations. Genomic DNA from the founders was sequenced to confirm the substitution of S18/20 and S68. *Twist1*^*S18I;20A/+*^ and *Twist1*^*S68A/+*^ heterozygous mice were crossed to obtain homozygous *Twist1*^*S18I;20A/S18I;20A*^ and homozygous *Twist1*^*S68A/S68A*^ embryos. These embryos were generated to determine the effects on the CNCC-derived craniofacial bone and cartilage, and the earliest time-point of detecting a craniofacial pathology. The animal work was approved by the Center for Laboratory Animal Medicine and Care committee at UT Health Houston under the Approved Animal protocol AWC-19-0045.

### Mouse handling, embryo extraction, and genotyping

Embryos were extracted based on the presence of a copulation plug and the number of days after the last known delivery. Pregnant females were euthanized with CO_2_ followed by cervical dislocation. Embryos were genotyped for *Twist1*^*fl/fl*^, *Twist1*^*+/*−^, *Twist1*^−*/*−^, *Twist1*^*fl/*−^, *Wnt1-Cre1, Wnt1-Cre2, Irf6*^−*/+*^, *Twist1*^*S18I;20A/S18I;20A*^ and *Twist1*^*S68A/S68A*^ alleles via DNA extraction, allele-specific PCR and gel electrophoresis. The ratio of genotype-to-phenotype of *Twist1* CKO and phosphor-incompetent mice was within the expected ratio for mouse.

### Histological and immunofluorescent staining

Embryos of wild-type, *Twist1*^−*/*−^, *Twist1*^*cko/*−^ and phospho-incompetent lines were collected for histological and immunofluorescence staining. Immunofluorescent staining was performed as previously described (Fakhouri et al., 2012). Briefly, mouse tissues were deparaffinized and rehydrated in a series of ethanol dilutions. The slides were boiled for 10 min in 10 mM sodium citrate buffer for antigen retrieval. Sections were blocked with anti-mouse IgG Fab fragment for 30 min, and then with 10% (v/v) normal goat serum and 1% BSA (v/v) in PBS for 1 h, then incubated overnight at 4°C with the following primary antibodies: mouse anti-AKT1/2/3 (1:150, Santa Cruz Biotechnology, sc-81434,), mouse anti-β-catenin (1:200, Santa Cruz Biotechnology, sc-7963), rabbit anti-β-catenin (1:150, Abcam, 16051), mouse anti-δ-catenin (1:150, BD Biosciences, 611537), rabbit anti-LRP2 (1:150, Abcam, ab76969), rabbit anti-RAB11b (1:150, Proteintech, 15903-I-AP), mouse anti-caludin 1 (1:200, Santa Cruz Biotechnology, sc-166338), mouse anti-N-cadherin (1:200, Santa Cruz Biotechnology, sc-59987), mouse anti-occludin (1:150, Santa Cruz Biotechnology, sc-133256), mouse anti-vimentin (1:150, Santa Cruz Biotechnology, sc-6260), mouse anti-E-cadherin (1:150, BD Biosciences, BD-610182), mouse anti-TWIST1 (1:200, Abcam, ab50887), SOX9 (1:200, Abcam, ab185966), mouse anti-ZO-1 (1:200, Santa Cruz Biotechnology, sc-33725), mouse anti-TFAP2α (1:50, DSHB, PCRP-TFAP2A-2C2), rabbit anti-CD73 (1:150, Abcam, ab133582), rabbit anti-CD206 (1:150, Abcam, ab64693), phalloidin (1:1000, Abcam, ab176757) and rabbit anti-IRF6 (1:500: Fakhouri et al., 2012). The secondary antibodies were goat anti-rabbit (1:150, A21429, Molecular Probes) and goat anti-mouse (1:150, A11029, Molecular Probes). We stained nuclei using DAPI (D3571, Invitrogen). An X-Cite Series 120Q laser and a CoolSnap HQ2 photometric camera (Andor Neo/Zyla) installed in a fluorescent microscope (Nikon Eclipse Ni) were used to capture images. For the immunofluorescence staining, sections from more than four embryos of each genotype, wild type and *Twist1*^*cko/*−^, were used for each embryonic time point tested.

### *Twist1* mRNA probe for *in situ* hybridization

The *Twist1* probe for *in situ* hybridization was designed to recognize a 0.8 kb region in the 3′ UTR region of mouse *Twist1*, starting at 188 bp and ending at 1062 bp after the *Twist1* coding sequence ends. This avoids any conserved regions homologous with other bHLH factors and encompasses the sequence of the shorter probe described by Wolf et al. (1991). Further details are provided in Fig. S3.

### Whole-mount embryo β-gal staining

*R26*^*Tm1*^ transgenic mice were used to perform X-gal staining for cell tracing in whole-mount embryos. Whole-mount embryo staining at E9.5, E11.5 and E15.5 was performed as previously described (Metwalli et al., 2018). Stained wild-type and *Twist1*^*cko/*−^ embryos were then visualized, analyzed and compared using a stereomicroscope and NIS Elements AR software. The X-gal staining was performed on more than three biological replicates of each genotype and embryonic stage tested.

### Skeletal staining

*Twist1* phospho-incompetent embryos at E16.5 were dissolved in 2% KOH until the skin dissolved, and then stained with Alcian Blue solution overnight for cartilage and Alizarin Red for bone, as previously described (Thompson et al., 2019). The embryos were then submerged in 10% glycerol and 1% KOH to remove excess stain and remaining tissues for 3-5 days.

### Neural tube explant excision and CNCC culture

E8.5-E9.5 embryos from the *Twist1*^*fl/*−^;*Irf6*^−*/+*^;*Wnt1-Cre2* crosses described previously were extracted from their amniotic sacs and maintained in an organ culture medium. Using microdissection instruments, E8.5-E9.5 embryos were removed from their amniotic sacs. Transverse cuts were made through the optic vesicle and first pharyngeal arch. After the removal of extraneous tissue, the mid and hindbrain regions were sectioned and cultured on collagen type I-coated petri dishes (10 µg/cm^2^) in alpha-MEM medium (10% fetal bovine serum, 100 U/ml penicillin G, 100 μg/ml streptomycin, and 1% non-essential and essential amino acids) and placed in a 5% CO_2_ incubator at 37°C overnight to allow for appropriate adherence.

### Time-lapse image acquisition and analysis

Time-lapse imaging of explants was performed using the A1R MP Confocal and Multiphoton Microscope (Nikon Instruments). Using overlaid contrast of red (TRITC), green (FITC) and blue (Hoechst) channels, fluorescence color changes from tissue explants were analyzed. Green fluorescence indicated the GFP reporter found only in CNCCs, while red fluorescence indicated all other tissues that express RFP, with Hoechst serving as a nuclei counterstain. Neural tubes from more than three biological replicates of each genotype were imaged in an environmentally controlled chamber maintained at 37°C and 5% CO_2_, and the migration of CNCCs was captured using automated acquisition over a 21 h period. The net migration of cell bodies over a 10 h period was calculated using IMARIS software (Bitplane). Parameters included a spot diameter and connected components algorithm used for cell tracking. Cells also were tracked frame by frame using the manual-tracking spot feature of IMARIS. About 16 wild-type and 22 *Twist1*^*cko/*−^ CNCCs were tracked. A two-tailed Mann–Whitney *U*-test was used with a significance level ****P<0.0001.

### Western blot, protein phosphorylation and RT-qPCR

We performed immunoblots to measure the quantitative levels of TWIST1 (ab50887), SNAIL2 (sc-166476), AKT1 (sc-81434), E-CAD (sc-8426), N-CAD (sc-59987), β-catenin (sc-7963) and WNT3 (sc-74537). mRNA expression of *Twist1* and *Irf6* in the hindbrain and first pharyngeal arch was also measured in three pooled biological and four technical replicates at E9.5. We also measured the mRNA level of these genes in addition to *Pi3k, Ck2, Erk1*, vimentin, occludin, *Msx1, Tfap2a, Hand2, Yap, Wnt1, Rhoa, Rhoa, Arhgap29, Sox2, Sox10, Hoxd10, Jag1, Src* and *Mtor*. The levels of phosphorylated and unphosphorylated forms of TWIST1 at S42, S68 and S127 were detected via immunoblot as previous described (Fakhouri et al., 2017; Hong et al., 2011). We received aliquots of rabbit polyclonal antibodies for TWIST1 P-S42 and P-S127 from Dr Brian A. Hemmings (Friedrich Miescher Institute for Biomedical Research, Basel, Switzerland; Vichalkovski et al., 2010). We purchased the monoclonal TWIST1 phospho-S68 from Abcam (ab187008). Monoclonal antibodies for TWIST1 (1:200, Abcam, ab50887) were used for immunofluorescence, immunoblotting and co-IP. We used the same antibodies previously described for E-CAD and β-catenin for immunofluorescence, western and co-IP. For the digestion with phosphatase enzyme, protein phosphatase 2A1 bovine (PP2A; Millipore-Sigma, P6993) was used to digest 100 µl total protein extracted from O9-1 CNCCs. About 10 µl PP2A in 50 mM TRIS-HCl buffer containing 2-mercaptoethanol was used and the solution was incubated at 30°C for 30 min and then analyzed by western blot using TWIST1 monoclonal antibodies (Abcam, ab50887).

### Sanger sequencing

Purified genomic DNA from mouse tail snips and purified PCR products were submitted for Sanger sequencing at Genewiz Company. We sequenced exon 1 of the Twist1-*null* and the two *Twist1* phospho-incompetent mouse lines for S18/20 and S68 regions.

### Co-IP assay for protein-protein interactions

We extracted total protein from mid and hindbrain tissues dissected from embryos at E8.5-E9.0. Tissues were ground with a plastic pestle on dry ice and the samples were frozen and thawed twice before centrifugation to remove undissolved materials. Purified total protein was incubated with Protein A/G conjugated to magnetic beads. We used monoclonal antibodies (Abs) for TWIST1 (1:200, Abcam, ab50887), E-CAD (1:150, Santa Cruz Biotechnology, sc-8426), δ-catenin (1:150, BD Biosciences, 611537) and β-catenin (1:150, Santa Cruz Biotechnology, sc-7963). We performed cellular fractionation for total protein extracted from E8.5 and E9.5 hindbrain tissues using a cytoplasmic buffer [50 mM Tris-HCl (pH 8), 137.5 mM NaCl, 0.05% Triton X-100, 10% glycerol, 5 mM EDTA and Protease Inhibitor Cocktail (Thermo Scientific)] and a nuclear buffer [50 mM Tris-HCl (pH 8), 137.5 mM NaCl, 0.1% Triton X-100, 0.5% SDS, 10% glycerol, 5 mM EDTA and Protease Inhibitor Cocktail (Thermo Scientific)]. The cytosolic and nuclear fractions were incubated individually with Protein A/G conjugated to magnetic beads for the co-IP assay.

### ChIP-PCR for TWIST1 binding to *Specc1l* putative regulatory elements

Chromatin immunoprecipitation (ChIP) was performed in tissues dissected from hindbrain and first pharyngeal arch. We used TWIST1 monoclonal antibodies to detect the binding to two putative enhancer elements within intron 1 and 2 of *Specc1l*. We also pulled down fragmented chromatins with IgG antibodies as a negative control for non-specific binding. The ChIP-PCR was performed as previously described (Kousa et al., 2019).

### Statistical analysis

The β-actin and ribosomal protein S16 (*Rps16*) were used for normalization of western blot and RT-qPCR data as internal controls, respectively. A two-tailed Student’s *t*-test analysis was applied for the statistical analysis between the wild-type control and *Twist1* CKO quantitative data. The difference in average mean value was considered statistically significant if *P*<0.05.

## Supplementary Material

Supplementary Material
