# Supplementary material for: TWIST1 interacts with β/δ-catenins during neural tube development and regulates fate transition in cranial neural crest cells
Source: Development. Author manuscript; Available in PMC 2025 Dec 24. (PMC9440756; doi:10.1242/dev.200068)
Supplement: Supplementary Material [file EMS211753-supplement-Supplementary_Material.pdf]

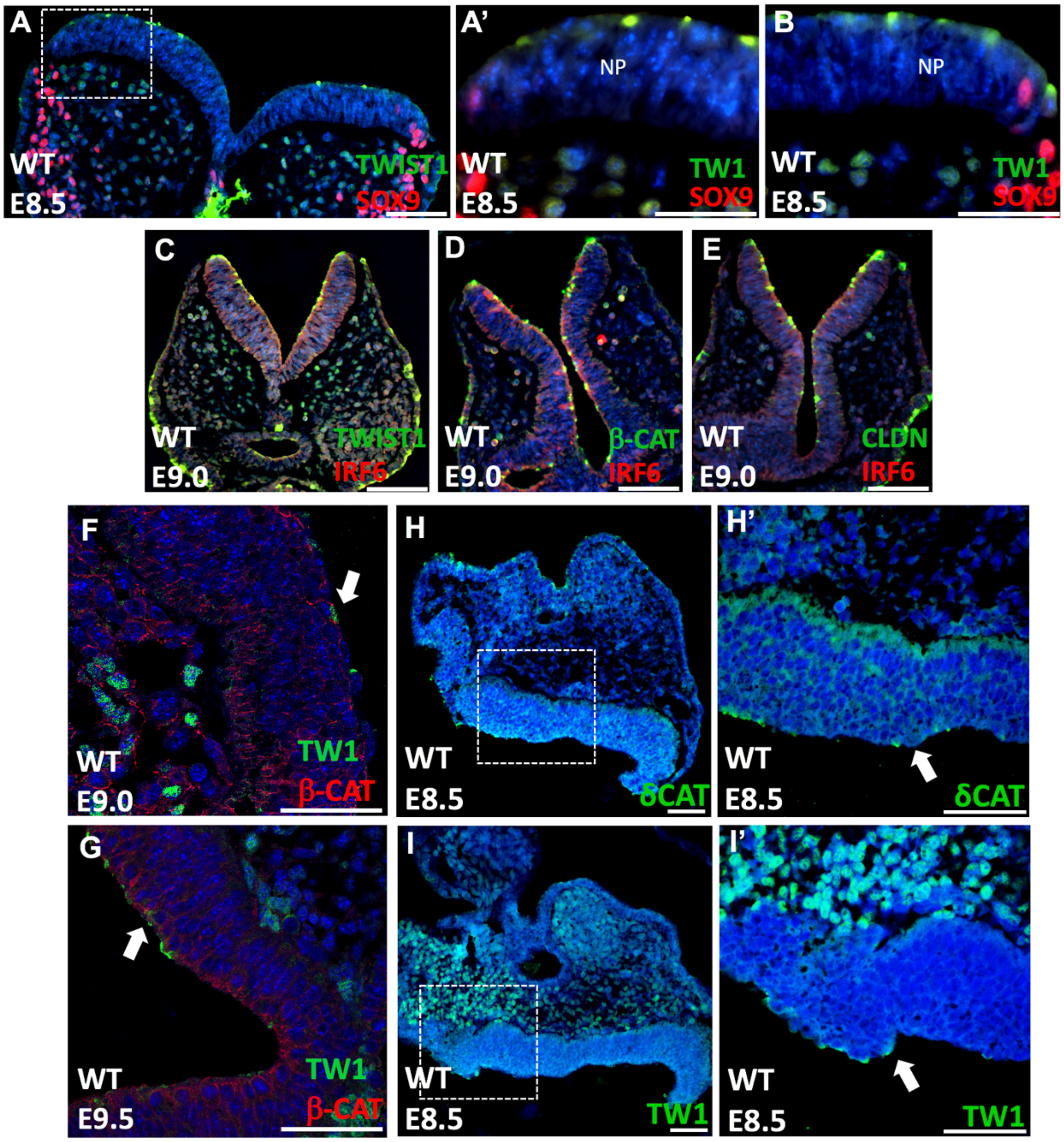

Fig. S1. Immunofluorescent staining of TWIST1, SOX9, CLAUDIN, IRF6, β-CATENIN, and δ-CATENIN during neural tube development. (A, A', B, C, D) TWIST1 is apically expressed at the dorsal side of the neural plate, similar to CLAUDIN and β-CATENIN. (E) TWIST1 is apically expressed in neural folds and at dorsal edges of neural folds. (F, G) Expression of CLAUDIN and β-CATENIN at the apical side of the neural folds at E9.0. (H, I) Co-expression of TWIST1 and β-CATENIN at the apical side of the neural folds and neural tube. (J-K') TWIST1 and δ-CATENIN are expressed in a similar pattern at the apical side of neural folds in consecutive sections.

Development • Supplementary information

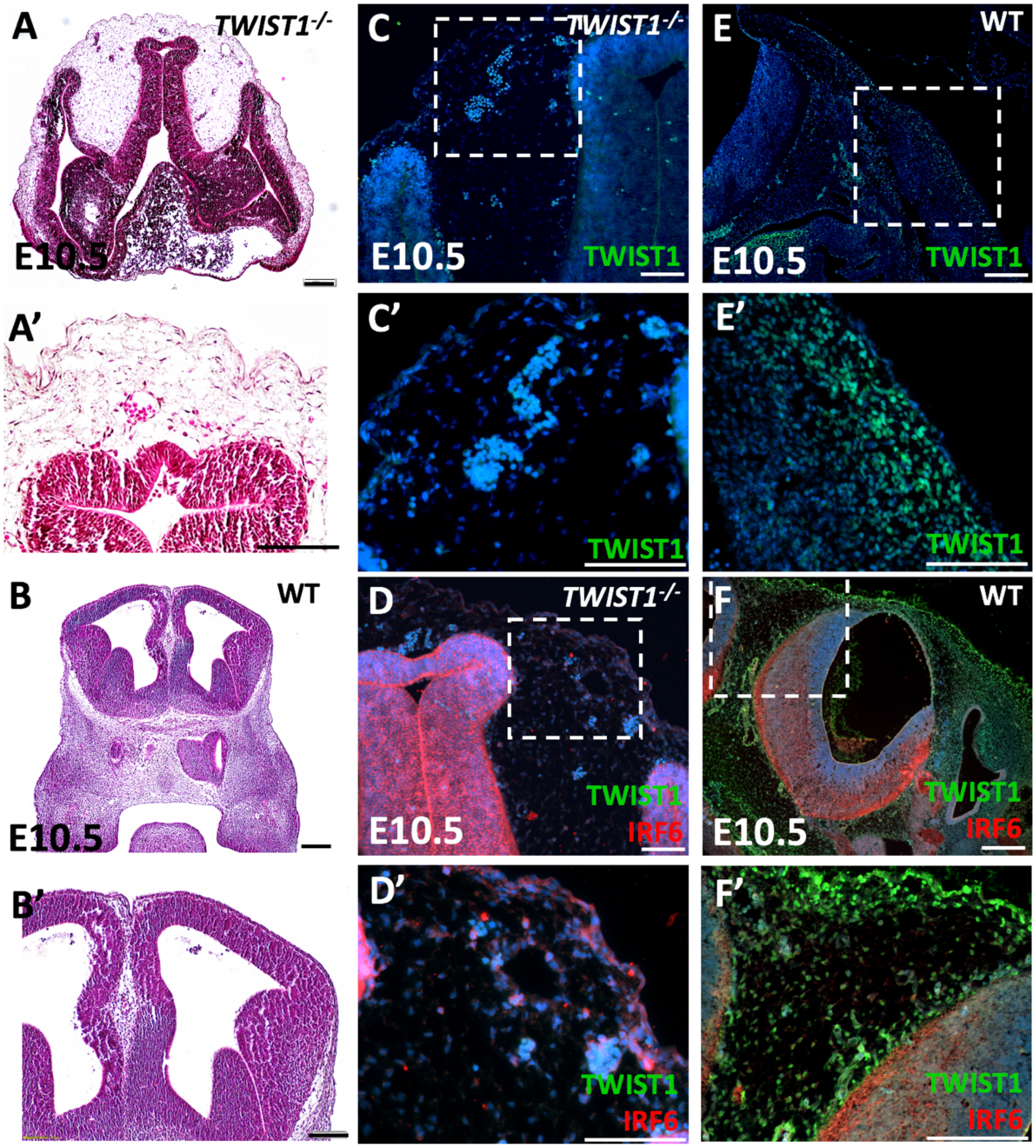

Fig. S2. Validation of anti-TWIST1 antibodies in Twist1 null embryonic tissues. (A, A') Histological staining of coronal section of *Twist1* null embryo showing the morphology of the frontal neural tube and complete loss of nasal and oral tissues at E10.5. (B, B') Histological staining of the coronal/transverse section of wild-type embryo shows the frontal neural tube morphology. (C, C') TWIST1 is not detected in *Twist1* null embryo using monoclonal anti-TWIST1 antibody by IF staining. (D, D') IRF6 is highly expressed in *Twist1* null embryo using dual staining for TWIST1 and IRF6. (E, E') TWIST1 is detected in migratory mesenchymal cells in comparable tissues of wild-type embryos using the same monoclonal antibody and conditions (F, F') IRF6 and TWIST1 are highly expressed in wild-type embryos using the same monoclonal antibody dual staining for TWIST1 and IRF6. Scale bars represent 100 μm.

Development • Supplementary information

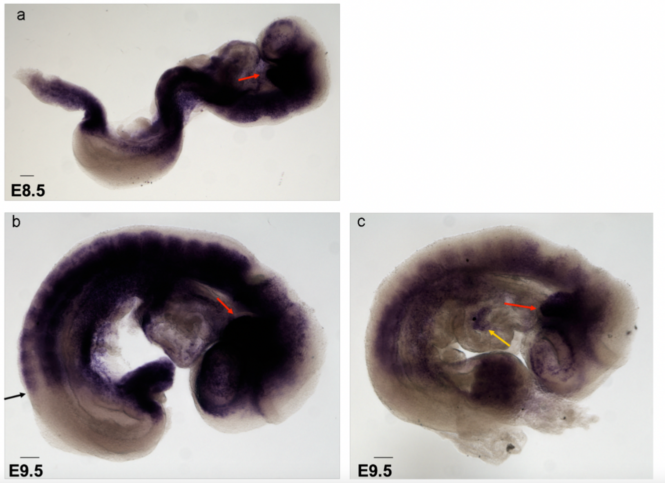

Fig. S3. Whole mount *in situ* hybridization for *Twist1* mRNA at E8.5 and E9.5 embryos. The positive staining is depicted in dark purple color in craniofacial, trunk and limb regions. To produce the RNA probe, BAC was extracted from clone bMQ-350m18 (GeneService). PCR primers were designed to amplify a 0.8 kb fragment in the 3' UTR region of *Twist1* gene. The fragment was amplified, subcloned into TOPO, and then sequenced using the M13 primers. Sequencing revealed that clone WOTO2 contained a correct insert which was used to produce a RNA probe complementary to *Twist1* mRNA using the T7 promoter site for transcription. The linearized and purified DNA plasmid was used as a template for T7 RNA polymerase (Roche, 10881767001) and the probe was labeled with the DIG-UTP for the *in situ* hybridization.

Development • Supplementary information

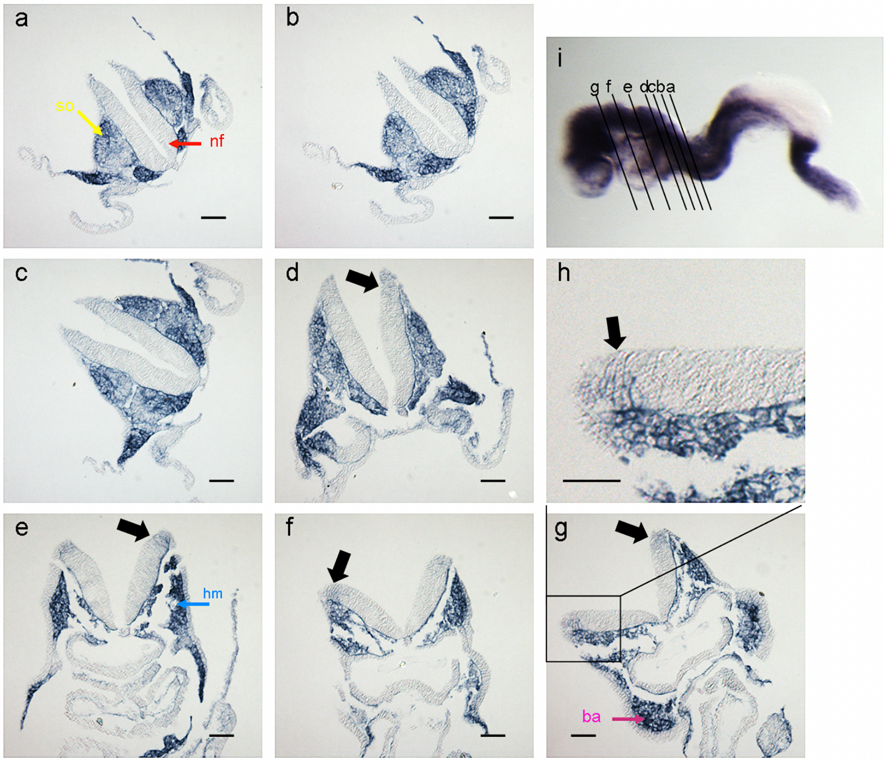

Fig. S4. ***Twist1* is weakly expressed in the dorsal neural plate at E8.5.** Whole mount *in situ* hybridization for *Twist1* of an E8.5 embryo sectioned from posterior to anterior (a to g) as shown in (i). Magnification of the box in (g) focusing on the dorsal neural tube (h). Neural fold (nf, red arrow), somite (so, yellow arrow), head mesenchyme (hm, blue arrow), branchial arch (ba, purple arrow). Scale bars represent 50 µm.

Development • Supplementary information

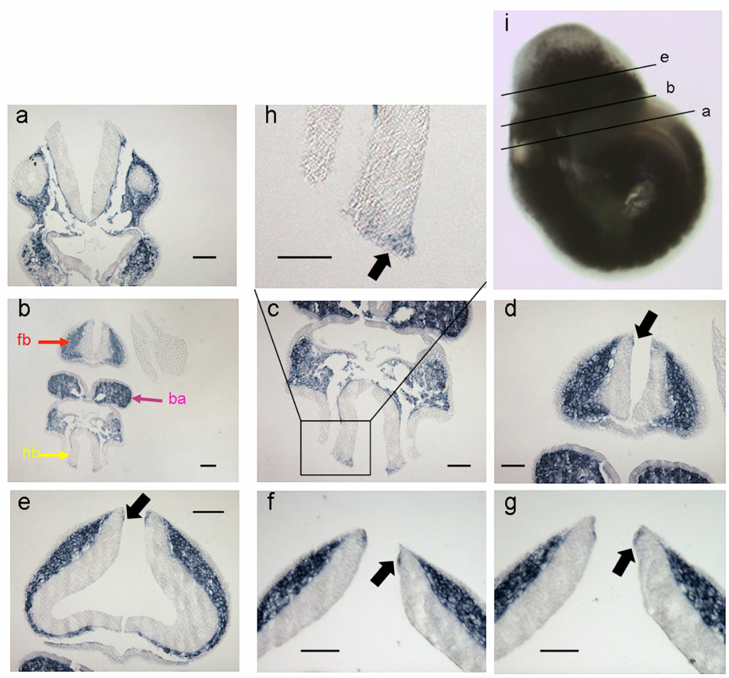

Fig. S5. ***Twist1* is weakly expressed in the dorsal neural fold at E9.5.** Whole mount *in situ* hybridization for *Twist1* of an E9.5 embryo sectioned from posterior to anterior (a to g) as shown in (i). Magnification of the box in (g) focusing on the dorsal neural tube. Forebrain (fb, red arrow), hindbrain (hb, yellow arrow), branchial arch (ba, purple arrow). Scale bars represent 50 μm.

Development • Supplementary information

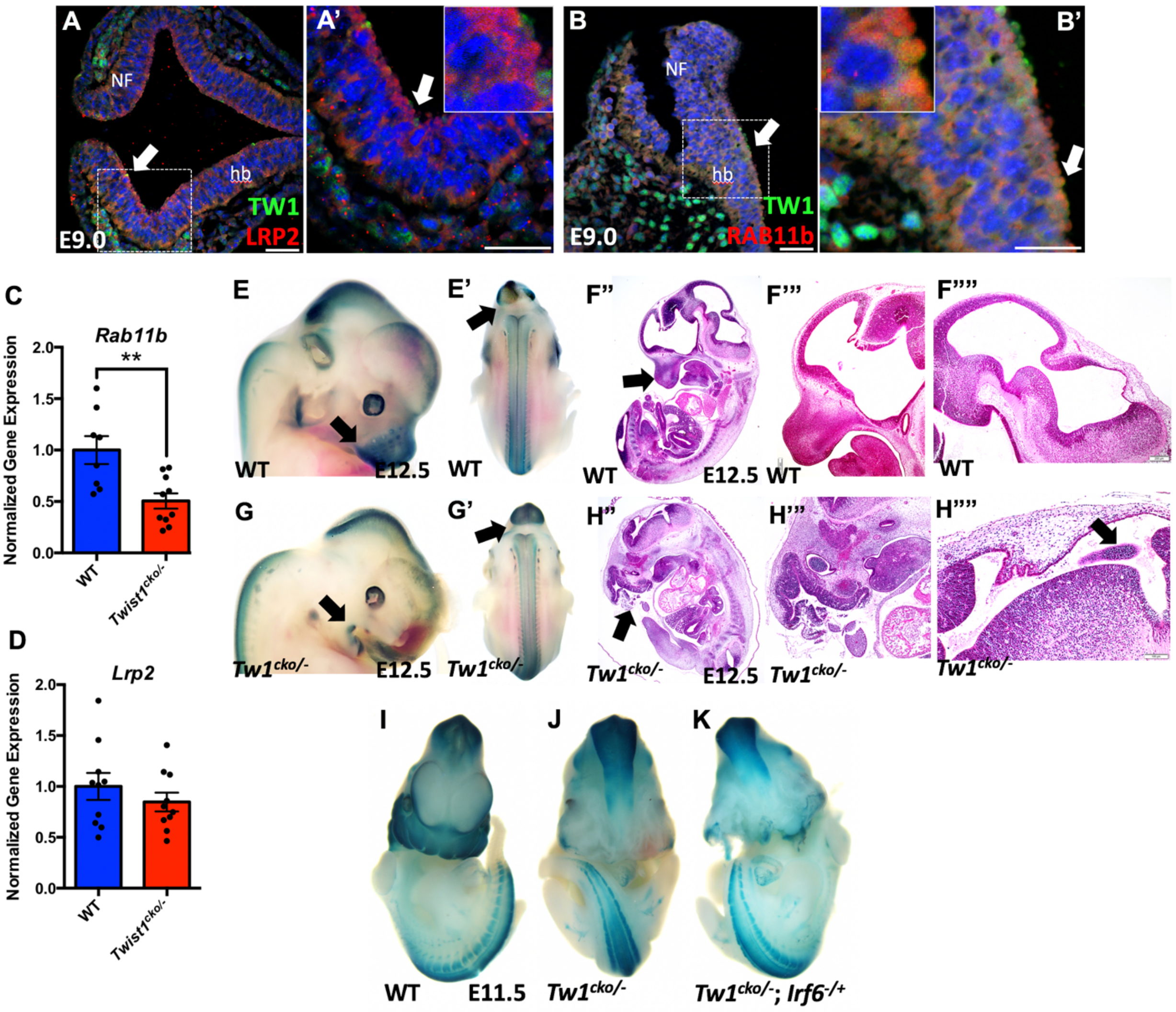

Fig. S6. (A, A') Dual immunostaining of TWIST1 in green and LRP2 in red are colocalized at the apical surface of neural folds. (B, B') Dual immunostaining of TWIST1 in green and endocytic marker RAB11b in red partially overlap in compartment vesicles at the apical side of neural fold. (C, D) Normalized expression level of *Lrp2* and *Rab11b* in wild type and *Twist1* CKO hindbrain tissues at E9.5. The whole mount X-gal and histological sections were staining in wild type and $Twist1^{cko/-}$ mouse embryos at

E12.5. (A, A') Whole mount X-gal of WT shows blue staining in frontonasal, jaw processes, midbrain, and trunk neural tube. (A"-A"") Histological staining shows normal brain development in wild type. (B, B') Whole mount X-gal of WT shows blue staining in frontonasal, jaw processes, midbrain, and trunk neural tube. (B"-B"") and severe brain abnormalities in $Twist1^{cko/-}$mouse embryos. (C-E) Whole mount X-gal staining of wild type, $Twist1^{cko/-}$, and $Twist1^{cko/-}$; $Irf6^{-/+}$ embryos at E11.5. Compound heterozygous embryo shows severe frontonasal deformities.

Development • Supplementary information

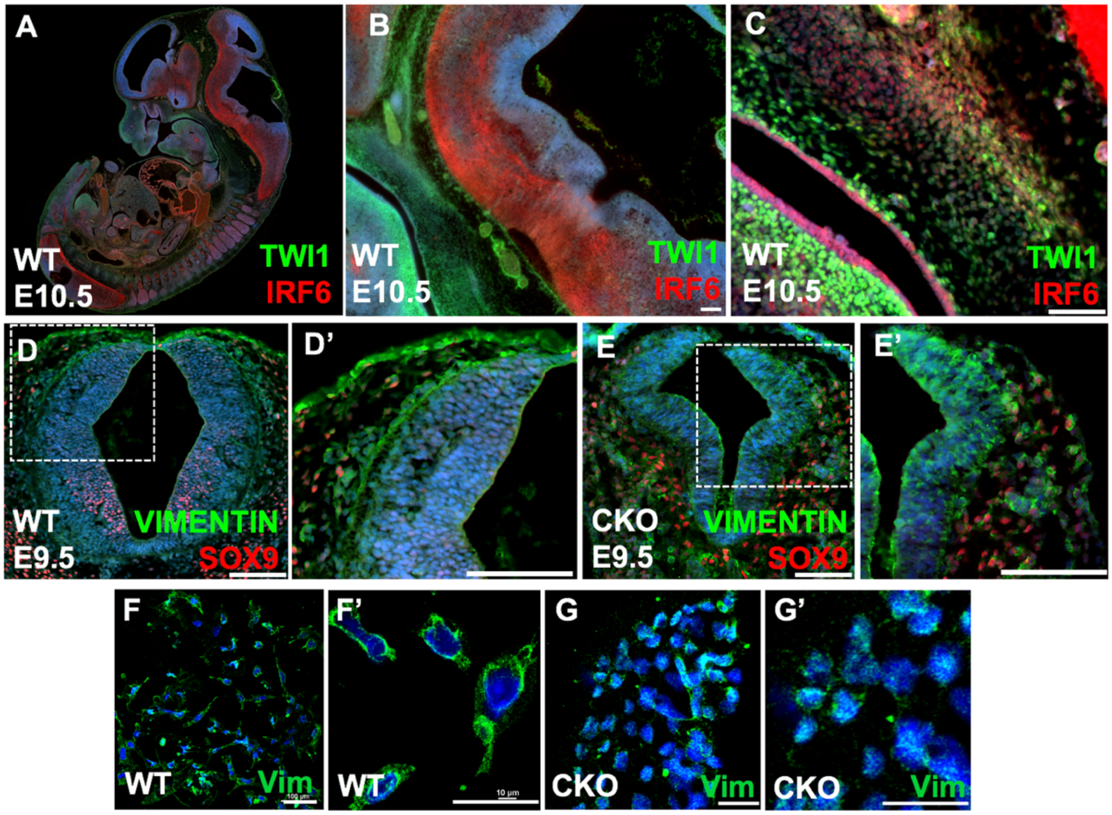

Fig. S7. Immunofluorescent staining of IRF6, TWIST1, SOX9, and VIEMENTIN in the neural tube and CNCCs. (A-C) IRF6 is highly expressed in neural tube and oral epithelium, while TWIST1 is highly expressed in migratory mesenchymal cells along the neural tube and in frontonasal and pharyngeal arches. (D-D') SOX9 is expressed in neuroectodermal progenitors of glial cells in neural tube, while VIEMNTIN is detected in migratory mesenchymal cells along the neural tube. (E, E') SOX9 was not observed in neural tube and VIEMNTIN was highly expressed in neural tube and partially detached cells along the neural tube. (F-G') VIEMNTIN is expressed in migratory CNCC emerged from neural tube explants of wild type and *Twist1*$^{cko/-}$ embryos.

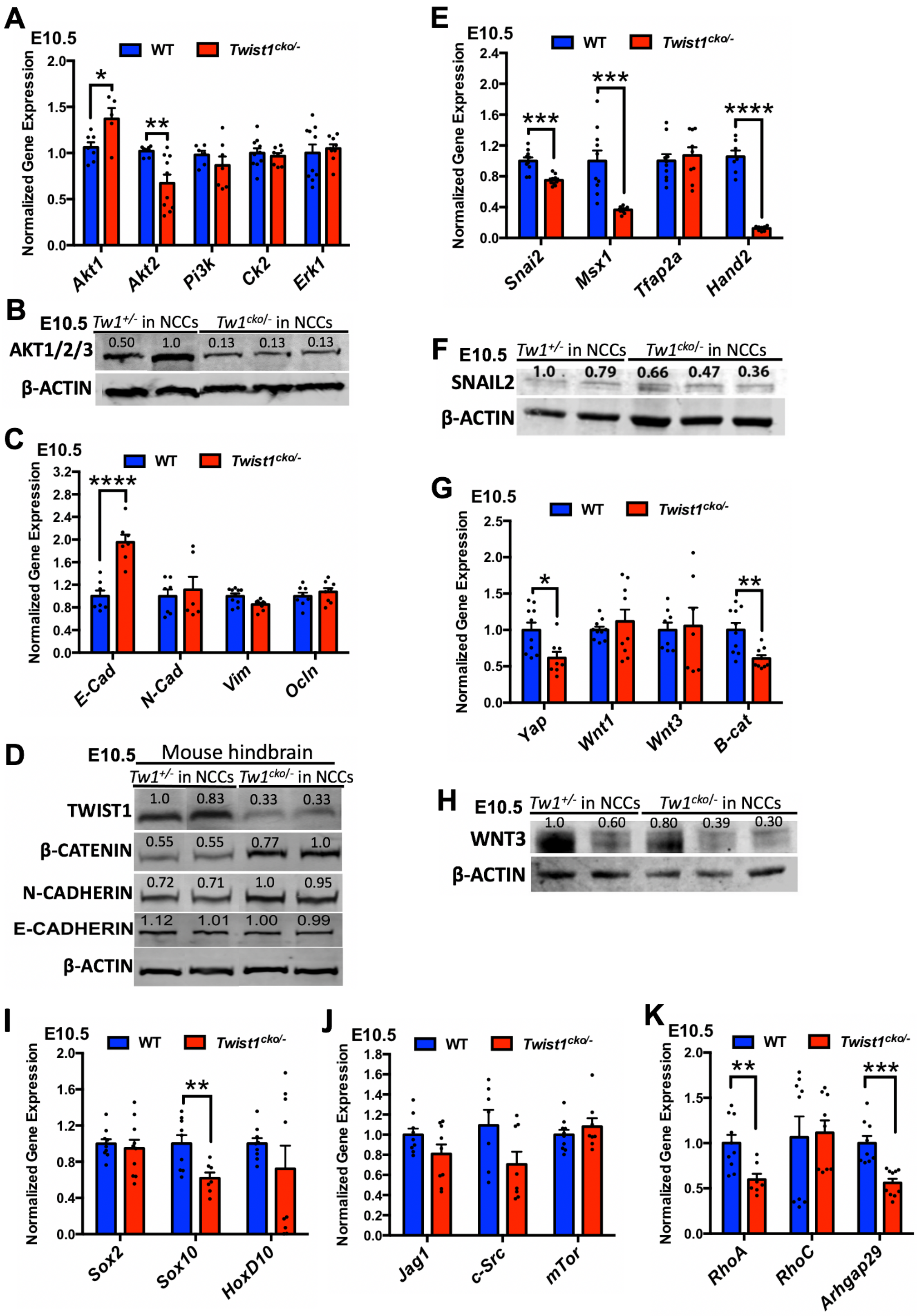
A
E10.5
WT
Twist1cko/-
Normalized Gene Expression
Akt1
Akt2
Pi3k
Ck2
Erk1
B
E10.5 Tw1+/- in NCCs Tw1cko/- in NCCs
AKT1/2/3
0.50 1.0 0.13 0.13 0.13
β-ACTIN
C
E10.5
E-Cad
N-Cad
Vim
Ocln
D
E10.5 Mouse hindbrain
Tw1+/- in NCCs Tw1cko/- in NCCs
TWIST1 1.0 0.83 0.33 0.33
β-CATENIN 0.55 0.55 0.77 1.0
N-CADHERIN 0.72 0.71 1.0 0.95
E-CADHERIN 1.12 1.01 1.00 0.99
β-ACTIN
E
Snai2
Msx1
Tfap2a
Hand2
F
E10.5 Tw1+/- in NCCs Tw1cko/- in NCCs
SNAIL2 1.0 0.79 0.66 0.47 0.36
β-ACTIN
G
Yap
Wnt1
Wnt3
B-cat
H
E10.5 Tw1+/- in NCCs Tw1cko/- in NCCs
WNT3 1.0 0.60 0.80 0.39 0.30
β-ACTIN
I
Sox2
Sox10
HoxD10
J
Jag1
c-Src
mTor
K
RhoA
RhoC
Arhgap29

Fig. S8. **Differentially expressed genes in *Twist1$^{cko/-}$ in-vivo*.** (A) Comparison of the expression of *Akt1*, *Akt2,* and *Pi3k, Ck2* and *Erk1* mRNA between WT and *Twist1$^{cko/-}$*. (B) Protein analysis by western showed a reduction in the kinase expression in mutant embryos at E10.5. (C) Comparison of the expression of *E-Cadherin* (adherens junction marker), *N-Cadherin*, *Vimentin* (mesenchymal marker), and *Occludin* mRNA in WT and *Twist1$^{cko/-}$*. (D) Protein analysis by western showed that adherens and tight junction proteins are increased in mutant embryos at E13.5. (E) Comparison of the expression of *Snai2, Msx1, Tfap2a and Hand2* mRNA between WT and *Twist1$^{cko/-}$*. (F) Protein analysis by western showed a reduction in the expression of SNAI2 transcription factor in mutant embryos at E10.5. (G) Comparison of the expression of *Yap*, *Wnt1*, *Wnt2*, *Wnt3* and *β-Catenin* mRNA in WT and *Twist1$^{cko/-}$*. (H) Protein analysis by western showed that WNT3 protein is decreased in mutant embryos at E13.5.

(I) mRNA expression of *Sox10* is significantly reduced in *Twist1$^{cko/-}$* tissues compared to WT. (J) mRNA expression of *Jag1*, *c-Src* and *mTor* was not altered in *Twist1$^{cko/-}$* compared to WT. (K) Comparison of the expression of *RhoA, RhoC, and Arhgap29* mRNA in WT and *Twist1$^{cko/-}$*. The level of *RhoA* and *Arhgap29* is significantly reduced in *Twist1$^{cko/-}$* embryos compared to WT.

Development • Supplementary information

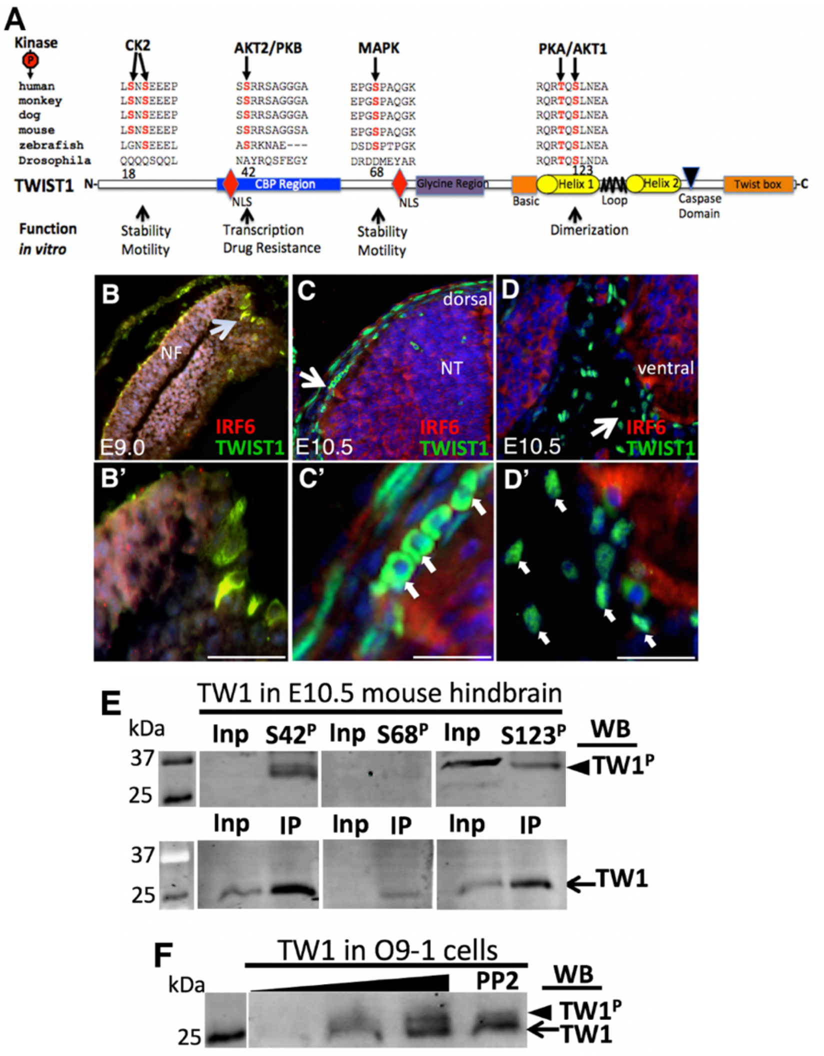

Fig. S9. **TWIST1 nuclear translocation and phosphorylation in CNCC**. (A) Mapping of phosphorylation sites within *TWIST1* functional domains and their respective kinases in cancer cell lines. TWIST1 has 6 phospho-residues based on *in vitro* cancer studies. All are highly conserved, particularly T121 and S123. (B-D') TWIST1 and IRF6 expression pattern in neural folds and CNCCs. (B-B') At E9.0, TWIST1 is expressed in the cytosol of dorsal lateral cells of the neural folds (NF). (C-C') At E10.5, TWIST1 is mostly cytosolic in pre-delamination CNCCs at the dorsal side of neural tube (NT). (D-D') TWIST1 becomes nuclear when the CNCCs detach and migrate toward the pharyngeal arches. (E) Immunoblot showing TWIST1 phosphorylated bands of S42, S68 and S123 (arrowhead) using polyclonal rabbit antibodies before immunoprecipitation (Input) and after enrichment (IP) using monoclonal TWIST1 antibodies (bottom blot, row) in WT mouse embryo at E10.5. (F) TWIST1 unphosphorylated (bottom arrow) and phosphorylated forms (arrowhead) are also highly expressed in O9-1 cells. Scale bars represent 50 µm.

Development • Supplementary information

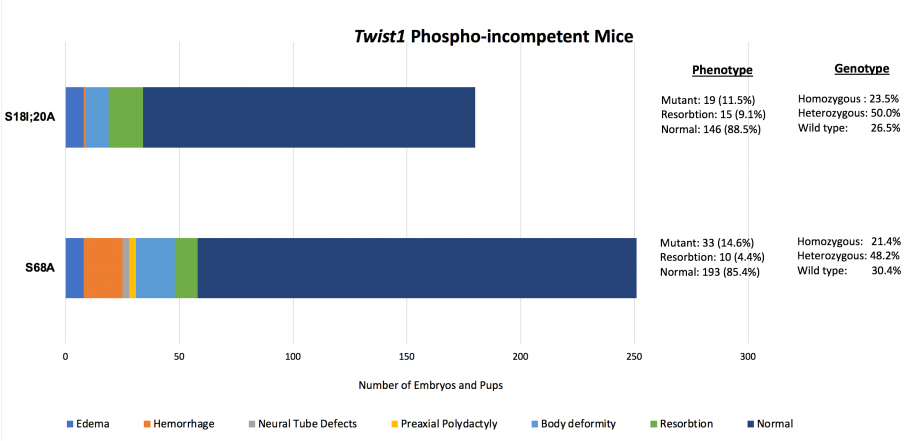

Fig. S10. The spectrum of craniofacial phenotype in the homozygous phospho-incompetent *Twist1*$^{S18;20A/S18;20A}$ and *Twist1*$^{S68A/S68A}$ embryos. Large numbers of embryos were collected from the two *Twist1* phospho-incompetent mouse lines to determine the ratio of genotype to phenotype and disease penetrance. The phenotype penetrance of the homozygous *Twist1*$^{S18I;20A/S18I;20A}$ and *Twist1*$^{S68A/S68A}$ mice was incomplete because the percentage of homozygous embryos and pups was 23.5% and 21.4%, respectively. However, the homozygous *Twist1*$^{S18I;20A/S18I;20A}$ and *Twist1*$^{S68A/S68A}$ embryos with phenotypes constitutes 11.5% and 14.6% of the total percentage, respectively, suggesting an incomplete penetrance of the phenotype. The percentage of mutant embryos is less than the estimated genotype percentage of the homozygous phospho-incompetent embryos.

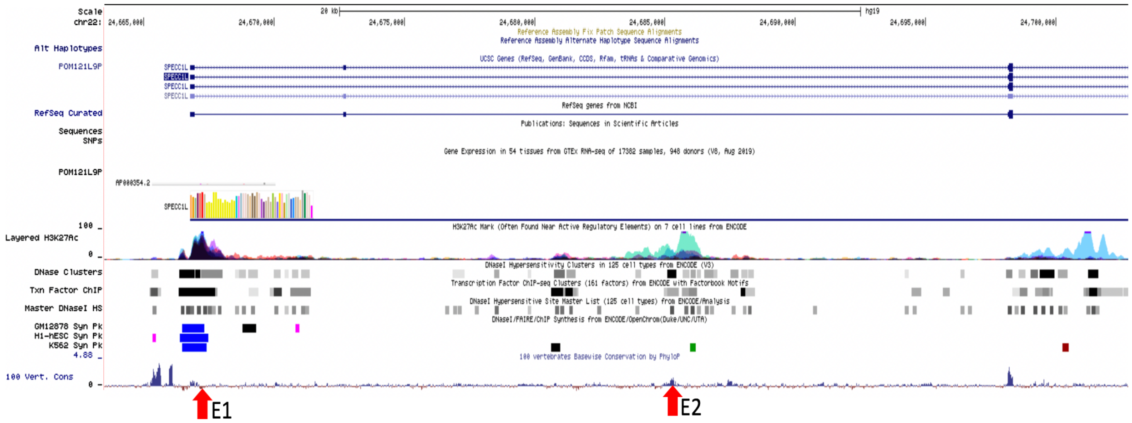

Fig. S11. A screenshot from UCSC Genome Browser showing the SPECC1L region. The two red arrows at the bottom indicate the genomic localization of putative enhancer elements E1 and E2 tested for TWIST1 binding. The two regions show enrichment for epigenetic enhancer signatures, including H3K27Ac, DNase I footprint, transcription factor signals, and conservation in 100 vertebrate species.

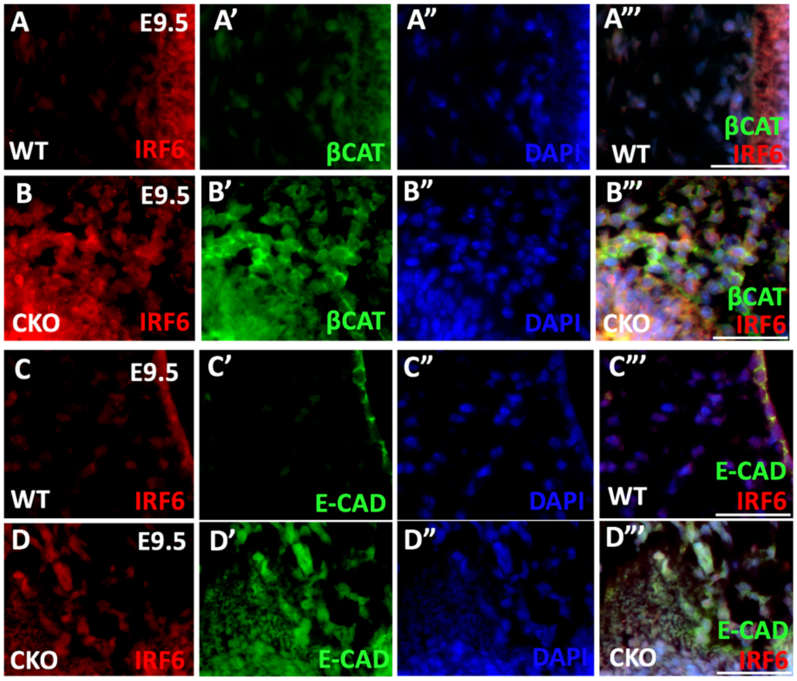

Fig. S12. Dual immunofluorescent staining of IRF6, β-CATENIN, and E-CAD in the neural tube and detached CNCCs. (A-A’”) IRF6 and β-CATENIN are highly expressed in the neural tube with no detectible expression of IRF6 in CNCCs. (B-B”’) IRF6 and β-CATENIN are highly expressed in neural tube and partially detached CNCCs. (C-C”’) Similarly, IRF6 and E-CAD are highly expressed in the neural tube with a weak signal of IRF6 in CNCCs. (D-D”’) IRF6 and E-CAD are highly expressed in the neural tube and in partially detached CNCCs.

Table S1. A list of genes and primers was used to quantify mRNA by RTqPCR, chromatin signal by ChIP-PCR, and cellular mRNA by *in situ* hybridization

**Genes**	**Primer Sequence**
mSpecc1l.qPCR.F	CCAAGGCCTTACCCGGTTT
mSpecc1l.qPCR.R	CCGGAGCAACAGGTCTTGAC
mPrrx1.qPCR.F	AACCTTCATCCTGGCATCCA
mPrrx1.qPCR.R	TGTGAAATTCCGCGAAGCT
mJag1.qPCR.F	TGGCCGAGGTCCTACACTTT
mJag1.qPCR.R	GCCGGCTAGGGTTTATCATG
mMsx1.qPCR.F	CCAGAAGATGCTCTGGTGAAG
mMsx1.qPCR.R	TTGGTCTTGTGCTTGCGTAG
mYap1.qPCR.F	GACTCGGAGACCGACTTGGA
mYap1.qPCR.R	AAGAAGGAGTCGGGCAGCTT
mArhgap29.qPCR.F	GCAAAATCGTCCATGTTTCGT
mArhgap29.qPCR.R	CCAACCTCCGCCTTTTTTC
mPi3k.qPCR.F	GGCCCAGATGCTCTATTTGC
mPi3k.qPCR.R	CCACGTAGCAGTCGGGAAAG
Wnt1-cre2.F	ACAGCGAACCATGCTGCCTG
Wnt1-cre2.R	CATGTCCATCAGGTTCTTGC
mSox2.qPCR.F	AAA GGA GAG AAG TTT GGA GCC
mSox2.qPCR.R	GGG CGA AGT GCA ATT GGG ATG
mSox10.qPCR.F	TCT GGA GGT TGC TGA ACG AAA
mSox10.qPCR.R	AGT CCG GAT GGT CCT TTT TGT
mHand2.qPCR.F	GGC CAA GGA CGA CCA GAA C
mHand2.qPCR.R	CGT TGC TGC TCA CTG TGC TT
mb-Catenin.qPCR.F	TGA CAC CTC CCA AGT CCT TT
mb-Catenin.qPCR.R	TTG CAT ACT GCC CGT CAA T
mC-Src.qPCR.F	CGG AAT GTG CTG GTG TCT GA
mC-Src.qPCR.R	CAG GCG CTG TCC ATT TGA CT
mRhoa.qPCR.F	CAT TGA CAG CCC TGA TAG TT

Development • Supplementary information

mRhoa.qPCR.R	TCG TCA TTC CGA AGG TCC TT
mRhoc.qPCR.F	TCG AAG TGG ATG GCA AGC A
mRhoc.qPCR.R	ACG TCA GTG TCC GGG TAG GA
mCk2.qPCR.F	TTT CTG GAC AAG CTG CTT CGA T
mCk2.qPCR.R	CAT TCG AGC CTG GTC CTT CA
mmTor.qPCR.F	CCA GGG GCT CCA ACG GCA AG
mmTor.qPCR.R	TCG TGG GGT CAG GCG GAA GA
mAkt2R.qPCR.F	CGG GCC TCT CCT TAT ACC CA
mAkt2R.qPCR.R	CTG CCC TGA GCT CAC TCA AG
mAkt1.qPCR.F	GGC TCA CCC AGT GAC AAC TCA
mAkt1.qPCR.R	CCC TTG CCC AGT AGC TTC AG
mHoxd10.qPCR.F	GTG GAC TGC TCC CGT CTT TG
mHoxd10.qPCR.R	TGT TCG GGT CTG TCC AAC TG
mEcad.qPCR.F	AATCCAACCCAGAGCTGCTC
mEcad.qPCR.R	TCATTTTCGGGGCAGCTGAT
mNcad.qPCR.F	CCATCCTGACAGACCCCAAC
mNcad.qPCR.R	GCACTTGGTTTTCTGCAGCA
mOcln.qPCR.F	GACTGGGCTGAACACTCCAA
mOcln.qPCR.R	ACATCACAGCTCACACCAGG
mVim.qPCR.F	GGATCAGCTCACCAACGACA
mVim.qPCR.R	TTCGGCTTCCTCTCTCTGGA
mSnai1.qPCR.F	CCTTCAGGCCACCTTCTTTG
mSnai1.qPCR.R	TGTGTCCAGTAACCACCCTG
mSnai2.qPCR.F	CCCTCCAAGTGCCTGTCTTA
mSnai2.qPCR.R	AGGCGTGGCTATTAACCGTA
mWnt1.qPCR.F	ATTCTTCTTCTGGGGTGGGG
mWnt1.qPCR.R	CCCGAGAGACAAGGAAAGGT
mWnt3.qPCR.F	TGTCCACCTTTACTACGCGT
mWnt3.qPCR.R	GAGCAGCCCATTCTTTCTGG
mIrf6_qRT_F1	AGTGTGGCCCAAAACAGAAC

Development • Supplementary information

mIrf6_qRT_R1	GGGTTGCTCACCGTCATAGT
mTwist1 qPCR-set2	CCCACTTTTTGACGAAGAAT
mTwist1 qPCR-set2	TGATCCCAGCGTTTTTATTT
mTfap2α RTqPCR F1	GAAGACTGCGAGGACCGTC
mTfap2α RTqPCR R1	GAAGTCGGCATTAGGGGTGTG
m18S.rRNA.qPCR.F	GCAATTATTCCCCATGAACG
m18s.rRNA.qPCR.R	GGCCTCACTAAACCATCCAA
mSpecc1l.ChIp.A.F	TAAAACAGAGACGGCCAGGT
mSpecc1l.ChIp.A.R	GGGTTTCTCTGTGTAGCCCT
mSpecc1l.ChIp.B.F	ACATATTTCCCCAGCCCCAA
mSpecc1l.ChIp.B.R	AGGGGTCCAAATAACTGCCA
ISH, PP015 Tw1.F<br>ISH, PP015 Tw1.R	CCCTGGCAAGCAGTTCAGTCC<br>TTCCTTCAGAGGAAGTATGGT
mLrp2. RTqPCR F1	GTCTAACCGCACTGTGATAGCC
mLrp2. RTqPCR R1	CGGAAGTTTCCTCCCAATGTGG
mRab11b. RTqPCR F1	TCAGATCTGGGACACTGCTG
mRab11b. RTqPCR R1	GGCTGAGGTCTCAATGAAGG

Development • Supplementary information
